# The plasmid-encoded Ipf and Klf fimbriae display different expression and varying roles in the virulence of *Salmonella enterica* serovar Infantis in mouse vs. avian hosts

**DOI:** 10.1371/journal.ppat.1006559

**Published:** 2017-08-17

**Authors:** Gili Aviv, Laura Elpers, Svetlana Mikhlin, Helit Cohen, Shaul Vitman Zilber, Guntram A. Grassl, Galia Rahav, Michael Hensel, Ohad Gal-Mor

**Affiliations:** 1 The Infectious Diseases Research Laboratory, Sheba Medical Center, Tel-Hashomer, Israel; 2 Department of Clinical Microbiology and Immunology, Sackler Faculty of Medicine, Tel-Aviv University, Tel-Aviv, Israel; 3 Abt. Mikrobiologie, Universität Osnabrück, Osnabrück, Germany; 4 Biovac Ltd., Or-Akiva, Israel; 5 Institute for Medical Microbiology and Hospital Epidemiology, Hannover Medical School, Hannover, Germany; 6 Sackler Faculty of Medicine, Tel-Aviv University, Tel-Aviv, Israel; University of California Davis School of Medicine, UNITED STATES

## Abstract

*Salmonella enterica* serovar Infantis is one of the prevalent *Salmonella* serovars worldwide. Different emergent clones of *S*. Infantis were shown to acquire the pESI virulence-resistance megaplasmid affecting its ecology and pathogenicity. Here, we studied two previously uncharacterized pESI-encoded chaperone-usher fimbriae, named Ipf and Klf. While Ipf homologs are rare and were found only in *S*. *enterica* subspecies diarizonae and subspecies VII, Klf is related to the known K88-Fae fimbria and *klf* clusters were identified in seven *S*. *enterica* subspecies I serovars, harboring interchanging alleles of the fimbria major subunit, KlfG. Regulation studies showed that the *klf* genes expression is negatively and positively controlled by the pESI-encoded regulators KlfL and KlfB, respectively, and are activated by the ancestral leucine-responsive regulator (Lrp). *ipf* genes are negatively regulated by Fur and activated by OmpR. Furthermore, induced expression of both *klf* and *ipf* clusters occurs under microaerobic conditions and at 41°C compared to 37°C, *in-vitro*. Consistent with these results, we demonstrate higher expression of *ipf* and *klf* in chicks compared to mice, characterized by physiological temperature of 41.2°C and 37°C, respectively. Interestingly, while Klf was dispensable for *S*. Infantis colonization in the mouse, Ipf was required for maximal colonization in the murine ileum. In contrast to these phenotypes in mice, both Klf and Ipf contributed to a restrained infection in chicks, where the absence of these fimbriae has led to moderately higher bacterial burden in the avian host. Taken together, these data suggest that physiological differences between host species, such as the body temperature, can confer differences in fimbriome expression, affecting *Salmonella* colonization and other host-pathogen interplays.

## Author summary

*Salmonella enterica* serovar Infantis is one of the prevalent serovars worldwide and is often associated with human gastroenteritis and asymptomatic persistence in poultry. Different emergent *S*. Infantis populations were shown to acquire a large virulence-resistance plasmid, named pESI. Here we imaged and investigated the phylogenetic distribution, regulation and the role in virulence of two previously uncharacterized pESI-encoded fimbriae, designated Klf and Ipf. We elucidate their complex regulatory network involving core (ancestral) and horizontally acquired regulators and demonstrated that their expression is significantly induced under microaerobic conditions and at 41°C compared to 37°C or the ambient temperature. Furthermore, we established that Klf and Ipf present a different expression profile and play a distinct role during mouse and chick infection, characterized by body temperature of 37°C and 41.2°C, respectively. Different composition of fimbriae or even allelic variation within a particular fimbria contributes to host tropism and can change the interaction of *Salmonella* with its host. Our work suggests additional mechanism, by which fimbriome expression is altered in response to the physiological temperature of different hosts. A distinct repertoire of expressed fimbriae is expected to affect *Salmonella* colonization and modify the host immune response to its infection.

## Introduction

The bacterial species *Salmonella enterica* (*S*. *enterica*) is a Gram-negative, highly ubiquitous pathogen that can infect a very wide range of animal and human hosts. This heterogeneous single species contains more than 2600 serovars that can differ in their adaptation to various hosts (host-specificity) and the disease they cause. For example, non-typhoidal serovars (NTS) such as *S*. *enterica* serovar Typhimurium (*S*. Typhimurium) or *S*. Enteritidis have a broad-host range and can infect many different animal species including reptiles, birds and mammals. In healthy humans, infection with NTS results in most cases in a localized self-limiting inflammation of the terminal ileum and colon, known as gastroenteritis. In contrast, *S*. Typhi or *S*. Paratyphi A can infect only humans (or high primates) and the disease they cause manifests as an invasive, life-threatening disease, called typhoid or enteric-fever (reviewed in [1,2]). Similarly, the avian-restricted serovars Gallinarum and Pullorum cause septicaemic diseases in poultry known as fowl typhoid and pullorum disease, respectively. Other *Salmonella* serovars or strains, although not fully host-restricted are well adapted to particular animal hosts. *S*. Choleraesuis, *S*. Dublin, *S*. Abortusovis and *S*. Typhimurium phage types DT2 and DT99 are often associated with swine, bovine, sheep, and pigeons, respectively [3–5].

One of the first steps in the establishment of a bacterial infection in any host is attachment to host tissues and colonization [6]. Intimate host-pathogen attachment is mediated by surface-exposed proteinaceous hair-like structures (pili) with adhesive properties, known as fimbriae that can bind specific glycoproteins or glycolipids host receptors [7,8]. Most of *Salmonella* fimbriae belong to the conserved chaperone-usher (CU) biogenesis pathway, named after the two proteins, required for the assembly of the pili, a periplasmic chaperone and an outer-membrane, pore-forming protein, termed usher. In addition to the major pilus subunit, CU fimbria may contain one or more minor protein subunits. Some of which are placed at the tip of the fimbrial rod and contain a lectin domain, providing adhesive properties to specific host receptors [9–11]. Thus, the composition of different fimbrial adhesins synergistically expressed by a specific strain or even polymorphism within a particular fimbria are thought to play a role in *Salmonella* host-tropism and adaptation [7,10,12–14].

The genes encoding the CU pathway are usually arranged within clusters encoding at least a major structural pilin subunit, a chaperone, and an usher protein. More complex fimbrial operons contain accessory genes encoding structural proteins such as minor fimbrial subunits, additional chaperones, or regulators. CU fimbrial clusters can be found either on the chromosome or on the plasmids of various Gram-negative bacteria [11,15].

Amongst more than 2600 *S*. *enterica* serovars known, *S*. Infantis is one of the most ubiquitous serovar worldwide. In the European Union, *S*. Infantis was ranked third in the prevalence order, following serovars Enteritidis and Typhimurium [16] and in the United States, *S*. Infantis was recently ordered sixth in the occurrence hierarchy [17]. In Israel, during 2008 to 2015, *S*. Infantis was the most predominant serovar, both in clinical (human) and poultry sources [18,19], suggesting that this serovar is highly adapted both to poultry and to the human hosts.

Previously, we showed that a rapid emergence of a new clone of *S*. Infantis in Israel has involved horizontal acquisition of a novel virulence-resistance megaplasmid, named pESI [18,19]. Recently, a related pESI-like plasmid was identified in an emergent *S*. Infantis populations in Italy [20] and in the USA [21], suggesting that pESI plays a similar role in globally emergent *S*. Infantis clones. In addition to antibiotic resistance and the potent yersiniabactin iron acquisition system, pESI encodes for two uncharacterized chaperone-usher fimbria clusters named *klf* and *ipf* [18].

Here, we studied environmental cues and the regulatory network controlling the expression of the Klf and Ipf fimbriae and characterized their role in *S*. Infantis virulence. We show the role of both core (Lrp, Fur and OmpR) and horizontally acquired (KlfB and KlfL) regulators in orchestrating these fimbriae expression and demonstrate that they are induced under microaerobic conditions and at the avian physiological temperature (41°C) compared to 37°C or the ambient temperature. Additionally, we establish that both fimbriae play a distinct role in the pathogenicity of *S*. Infantis in the mammalian vs. the avian hosts. We propose that these differences affect *S*. Infantis colonization and contribute to variations in host-pathogen interactions.

## Results

### Genetic organization, classification and phylogenetic distribution of the Ipf and Klf fimbriae

**Fig 1:**
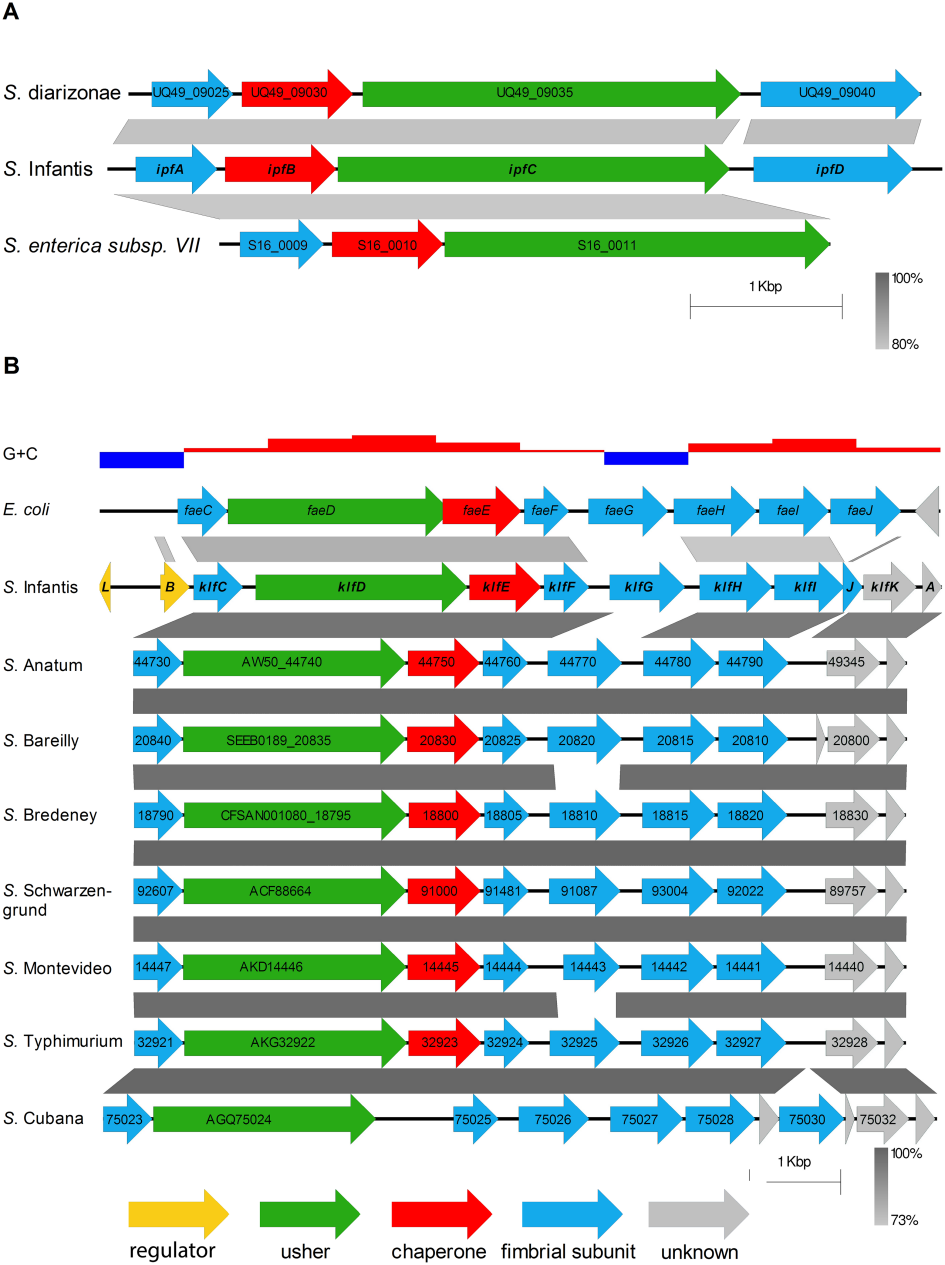
Genetic organization and phylogenetic distribution of the *ipf* and *klf* clusters. The pESI-encoded *ipf* and *klf* gene clusters were compared using the Easyfig tool with homologous clusters found in the nr database. Nucleotide comparison between the related clusters is illustrated by the shades of grey that indicate the degree of sequence homology (in %). Gaps in the grey areas point to the lack of sequence similarity, and protein functions (regulator, usher, chaperone and fimbrial subunit) are color-coded. **(A)** The *ipf* cluster encodes four ORFs: IpfA, a fimbrial protein; IpfB, the fimbrial chaperone; IpfC, the fimbrial usher; and IpfD, a putative fimbrial adhesin. Homologs of the *ipf* cluster were found in *Salmonella enterica* subsp. diarizonae strain 11–01854 (GenBank: CP011292.1) and in *Salmonella enterica* subsp. VII integrative and conjugative element ICESe3 region, strain SARC16 (sequence ID: FN298495.1). **(B)** The *klf* cluster in *S*. Infantis 119944 contains 12 ORFs encoding the major fimbrial subunit (KlfG), usher (KlfD), chaperone (KlfE) and four minor subunits (KlfC, KlfF, KlfH and KlfI). KlfJ, KlfK and KlfA have unknown function and KlfL and KlfB are unique to pESI and function as regulators (see Fig 8). Similarity to the *fae* cluster in enterotoxigenic *E*. *coli* (GenBank: CP002730) is shown at the upper line of the alignment. Homologous clusters among *S*. *enterica* serovars were found in *S*. Anatum str. USDA-ARS-USMARC-1735 (GenBank: CP007584.2); *S*. Bareilly str. CFSAN000189 (GenBank: CP006053); *S*. Bredeney str. CFSAN001080 (GenBank: CP007533); *S*. Schwarzengrund str. CVM19633 (GenBank: CP001127.1); *S*. Montevideo str. USDA-ARS-USMARC-1921 (GenBank: CP007540.1); *S*. Typhimurium strain FORC_015 (GenBank: CP011365); and *S*. Cubana str. CFSAN002050 (GenBank: CP006055.1). The G+C content is illustrated by the blue-red histogram, at the top of the panel, while G+C > 50% is shown in red and G+C < 50% is shown in blue.

The virulence megaplasmid, pESI encodes for two independent chaperone-usher fimbriae, one is related to the known K88-Fae fimbria and therefore named K88-like fimbria (Klf), while the other was designated Ipf (standing for Infantis plasmid encoded fimbria) [18]. The *ipf* fimbrial cluster belongs to the γ_1_-fimbrial clade [15], occupies 5.1 kb and contains four ORFs encoding a major fimbrial subunit (IpfA), a chaperone (IpfB), an usher (IpfC), and a putative adhesin (IpfD). Interestingly, nucleotide-nucleotide BLAST (blastn) search against the NCBI nr database showed a very limited distribution of the *ipf* cluster that was found, besides pESI, only outside of subspecies enterica (ssp. I), in the genome of *Salmonella enterica* ssp. diarizonae (ssp. IIIb) and in an integrative and conjugative element ICESe3 of *Salmonella enterica* ssp. VII (S1 Table and Fig 1A).

**Fig 2:**
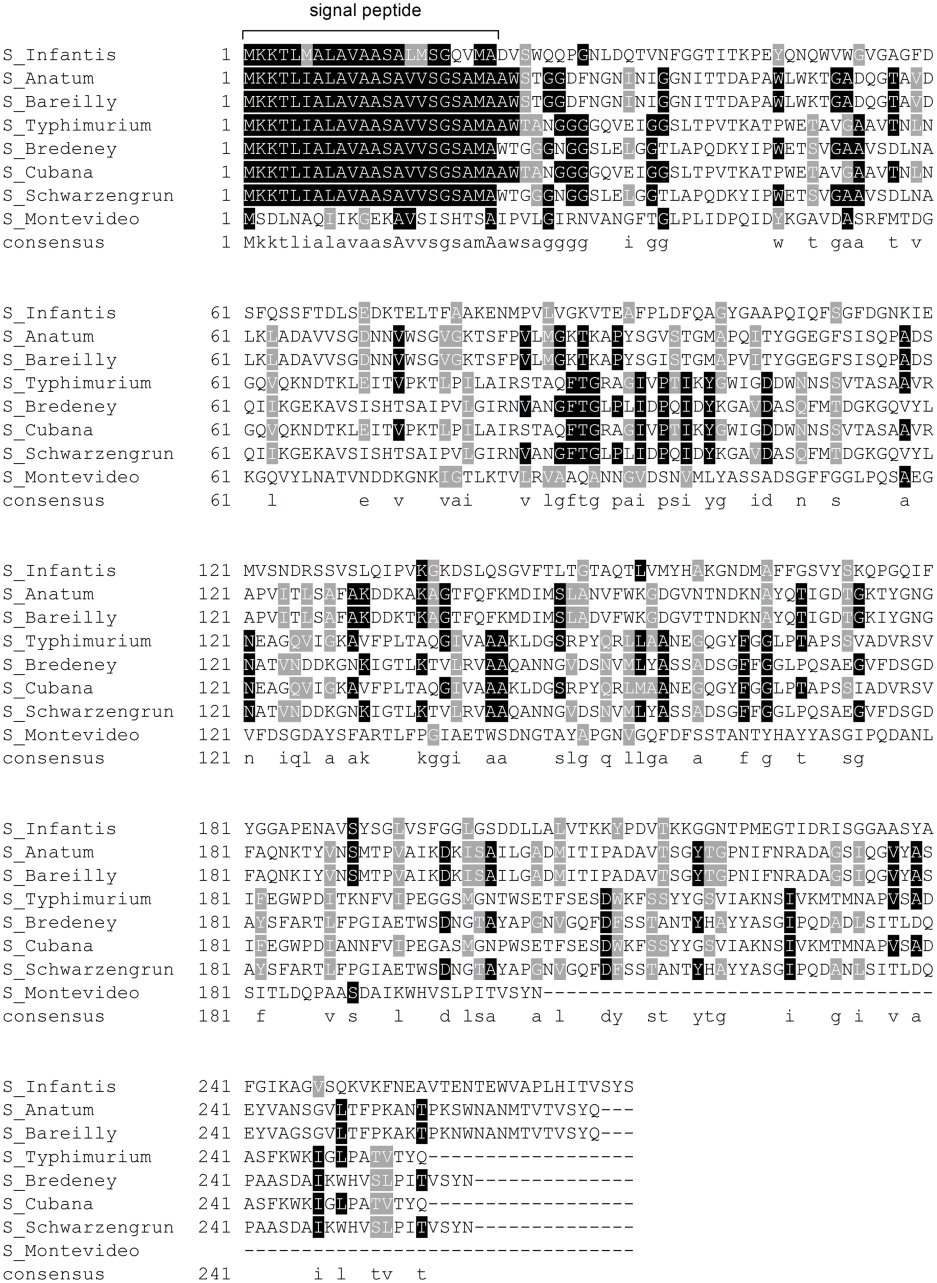
Klf homologs harbor different alleles of the fimbrial major subunit KlfG. Multiple sequence alignment of the KlfG from the eight *klf* clusters shown in Fig 1B was performed using CLUSTALW and BOXSHADE tools. Identical amino acids are shown in black and similar amino acids are shown in grey. The conserved signal peptide sequence preceding the Sec cleavage site was predicted by the SignalP 4.1 program and is indicated at the N-terminus of the proteins.

The second pESI-encoded fimbria is related to the K88-Fae fimbria and belongs to the κ-fimbrial clade [15]. Enterotoxigenic *E*. *coli* (ETEC) strains expressing the K88 fimbria are long known to cause diarrheal disease in piglets and calves [22,23]. Nevertheless, since the related fimbrial cluster in pESI shows significant rearrangements and the homologous proteins present less than 90% identity to the corresponding K88 subunits in *E*. *coli* (S2 Table and Fig 1B), we renamed this pESI-encoded cluster *klf* as mentioned above. The *klf* fimbrial cluster is 9.3 kb long and consists of 12 putative ORFs (Fig 1B) encoding an usher protein (KlfD), chaperone (KlfE), major fimbrial subunit (KlfG) and four minor fimbrial subunits (KlfC, KlfF, KlfH and KlfI). At the 3' end of this cluster, we identified three hypothetical proteins with unknown function: KlfJ that might be truncated, KlfK and KlfA. In addition, two putative regulatory proteins (KlfL and KlfB) were annotated at the 5'-end of the *klf* cluster. In contrast to the rare distribution of the *ipf* cluster, *klf* homologs were found in several ssp. I *Salmonella* genomes, including in strains from serovars Anatum, Bareilly, Bredeney, Typhimurium, Cubana, Schwarzengrund, and Montevideo. Yet, the 5'-region of 1091 bp, containing the genes *klfL* and *klfB* appeared unique to the pESI-*klf* cluster and was not found in any other related cluster. Furthermore, while KlfC, KlfD, KlfE, KlfF, KlfH, KlfI, KlfK and KlfA are highly conserved among *S*. *enterica* Klf homologs, KlfG corresponding proteins, although presented similar size, were greatly diverse in their sequence (S3 Table). In fact, the only KlfG conserved domain was its N-terminus, containing the signal peptide sequence, required for protein export via the Sec pathway (Fig 2).

These results suggested that the *klf* cluster was subjected to significant genetic rearrangements including an insertion of the *klfL-klfB* fragment and recombination in the *klfG* gene. This possibility was further supported by the G+C profile of these two regions, showing a distinct G+C content of 38% and 42% for the *klfL*-*klfB* and *klfG* regions, respectively, compared to 61% G+C composition typical to the rest of the *klf* cluster (see the top histogram of Fig 1B), or 52% characterizing the entire *S*. Infantis genome.

### The *ipf* and *klf* clusters encode structurally-distinct fimbriae

**Fig 3:**
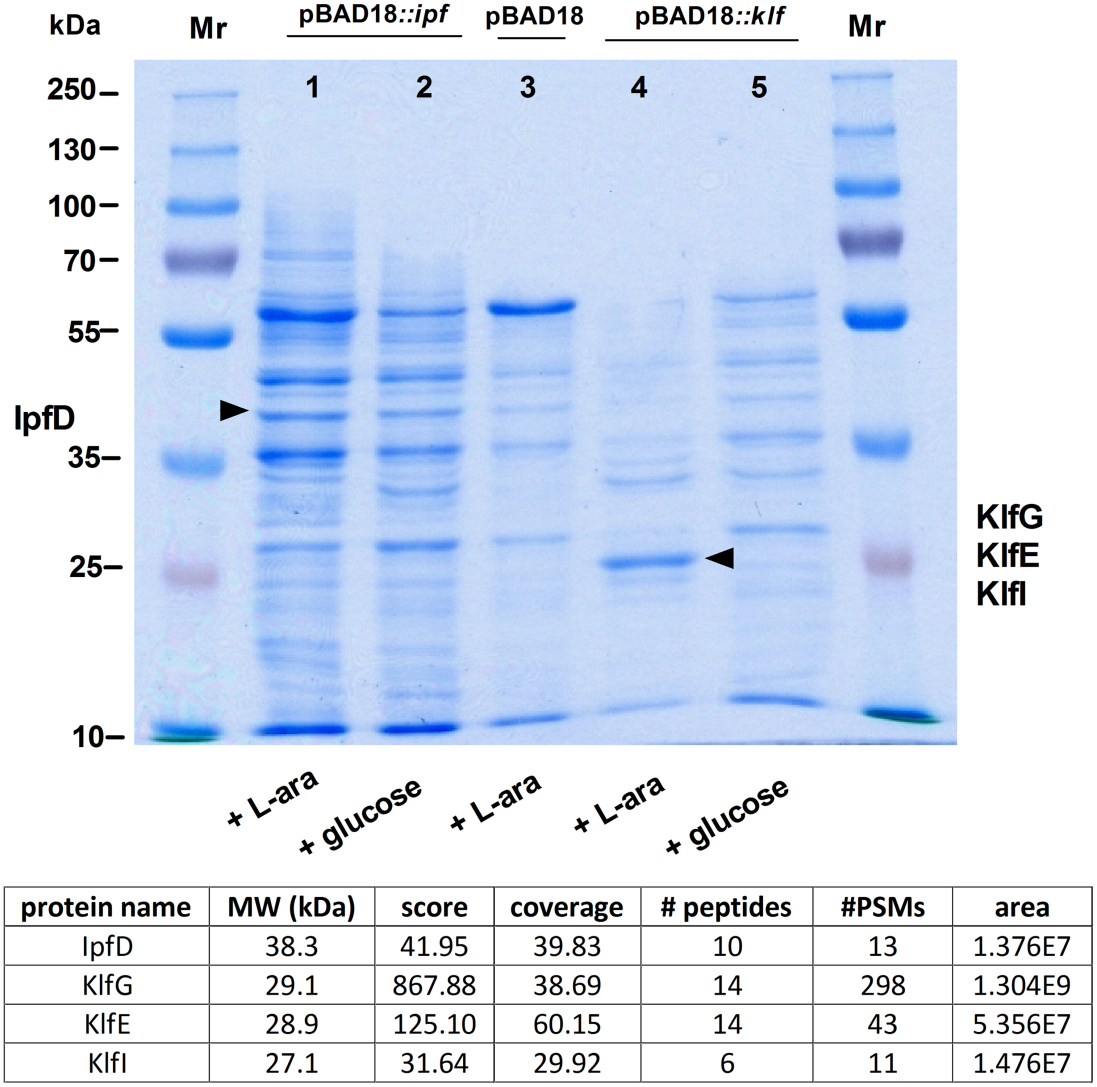
Heterologous expression of Ipf and Klf in surrogate *E*. *coli* cells. Non-fimbriated *E*. *coli* strain ORN172 carrying the entire *ipf* operon (pBAD18::*ipf*, lanes 1 and 2), the *klf* operon (pBAD18::*klf*, lanes 4 and 5), or the empty vector (pBAD18, lane 3) that was used as a negative control was grown in N-minimal medium supplemented with 50 mM L-arabinose (inducing conditions) or 1 M glucose (suppressing conditions). Cultures supernatants that were enriched with surface structures were collected after a shearing treatment, subjected to TCA precipitation and separated on a 12% SDS-PAGE. Arrows show the bands that were isolated from the gel and identified by LC-MS/MS. The proteins that were detected by LC-MS/MS are summarized in the table below. The score value presents the cumulative protein score based on summing the ion scores of the unique peptides identified for that protein. Coverage displays the percentage of the protein sequence covered by the identified peptides. PSMs show the total number of identified peptide sequences (peptide spectrum matches) for the protein, including those redundantly identified. The area displays the average area of the three unique peptides with the largest peak area.

To gain further insights into the biology of Ipf and Klf fimbriae, both clusters were cloned under an arabinose-inducible promoter in pBAD18 and introduced into a non-fimbriated *E*. *coli* ORN172 strain. Cultures that were grown in minimal medium to the late logarithmic phase in the presence of arabinose (as inducer) or glucose (as suppressant) were subjected to shearing by a shaft homogenizer. Filtered bacterial cell supernatant, enriched with surface exposed macromolecules was precipitated by trichloroacetic acid (TCA) and protein precipitates were separated by SDS-PAGE. Protein bands with the expected molecular weight of the fimbriae subunits, which were enriched in the arabinose-induced cultures were isolated from the acrylamide gel and analyzed by LC-MS/MS. This analysis successfully identified the presence of IpfD (the putative adhesive lectin) and three subunits of the Klf fimbria including KlfG (major subunit), KlfE (chaperone) and KlfI (minor subunit) (Fig 3), indicating successful expression of these fimbrial components in the heterologous bacterial host.

**Fig 4:**
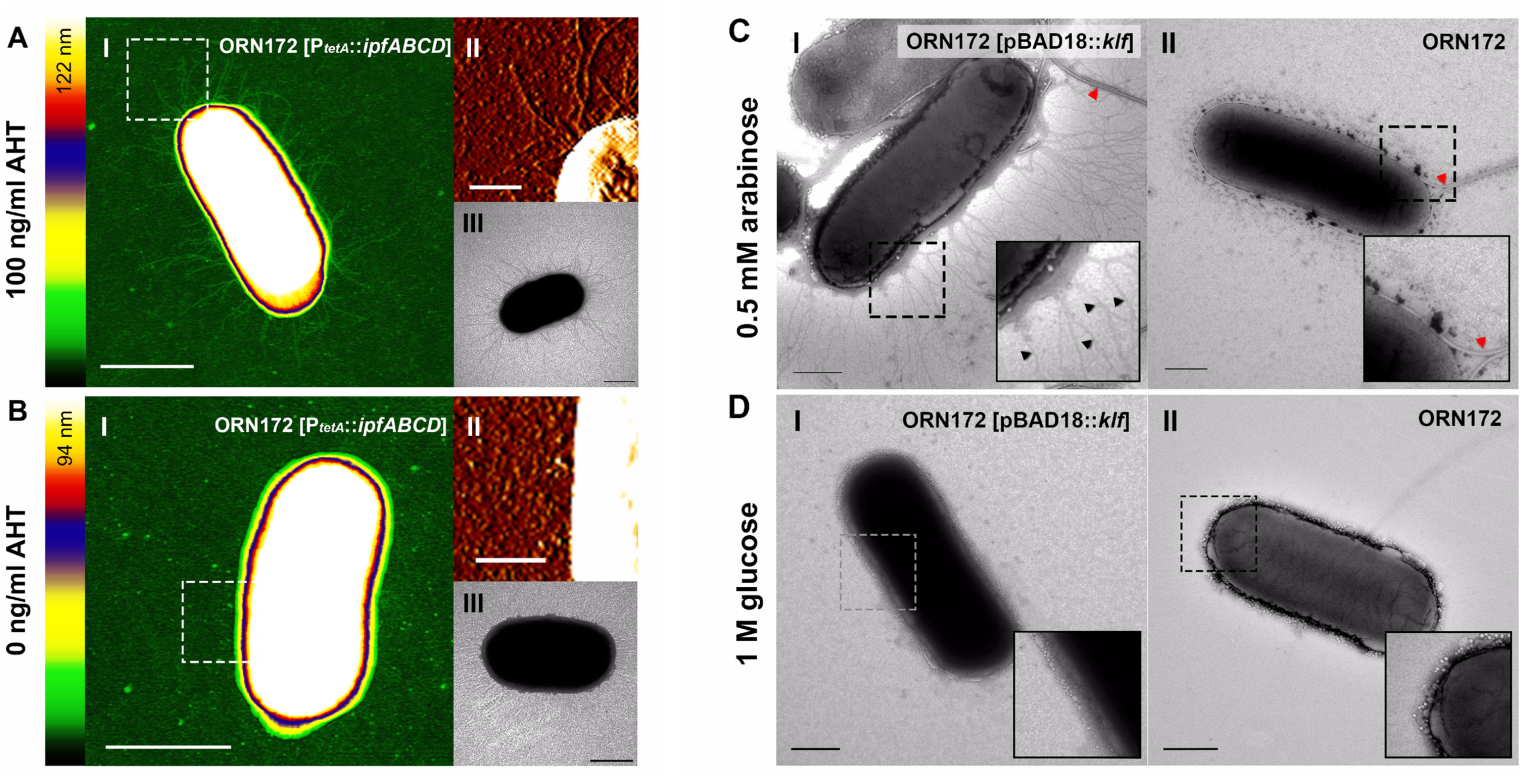
The *ipf* and *klf* clusters encode structurally-distinct fimbriae. **(A, B)**: Fimbriae-less *E*. *coli* ORN172 expressing *ipfABCD* under the tetracycline-inducible promoter (P*tetA*) were grown in N-minimal medium supplemented with 100 ng/ml anhydrotetracycline (AHT; **A**) or in the absence of the inducer (**B**). Cultures harboring *ipfABCD* were visualized by AFM and TEM. Panels **A** and **B** show an AFM height image (I), an enlarged AFM deflection image (II) and a TEM image (III). Highlighted box in (I) indicate the area shown in AFM deflection image (II). Color bar indicates the Z-range. Scale bars, 1 μm (I), 0.25 μm (II), 1 μm (III). **(C, D)** *E*. *coli* ORN172 harboring *klfBCDEFGHIJKA* under control of an arabinose-inducible promoter (P*ara*) were grown in N-minimal medium supplemented with 0.5 mM arabinose (**C**), or 1 M glucose (**D**). Bacterial cells were negatively stained with 0.5% phosphotungstic acid (PTA) and imaged by TEM. Scale bars, 500 nm. Black and red arrows indicate the Klf fimbriae or flagella, respectively.

*E*. *coli* ORN172 expressing *klfBCDEFGHIJKA* under the arabinose promoter (P*ara*) and *ipfABCD* under the tetracycline-inducible promoter (P*tetA*) were next imaged using transmission electron microscopy (TEM) and atomic force microscopy (AFM). Using these high-resolution microscopy techniques, we were able to image the Ipf fimbriae, which appear as short and thin pili of about 1.5 nm thickness (Fig 4A), and the Klf fimbriae that create a complex net of thin webs around the bacteria envelope (Fig 4CI). These distinct apparatuses were absent from the non-inducible cultures (Fig 4B and 4DI) and demonstrated structurally diverse fimbriae encoded from the pESI megaplasmid.

### *ipf* and *klf* are induced under microaerobiosis and at the avian physiological temperature

**Fig 5:**
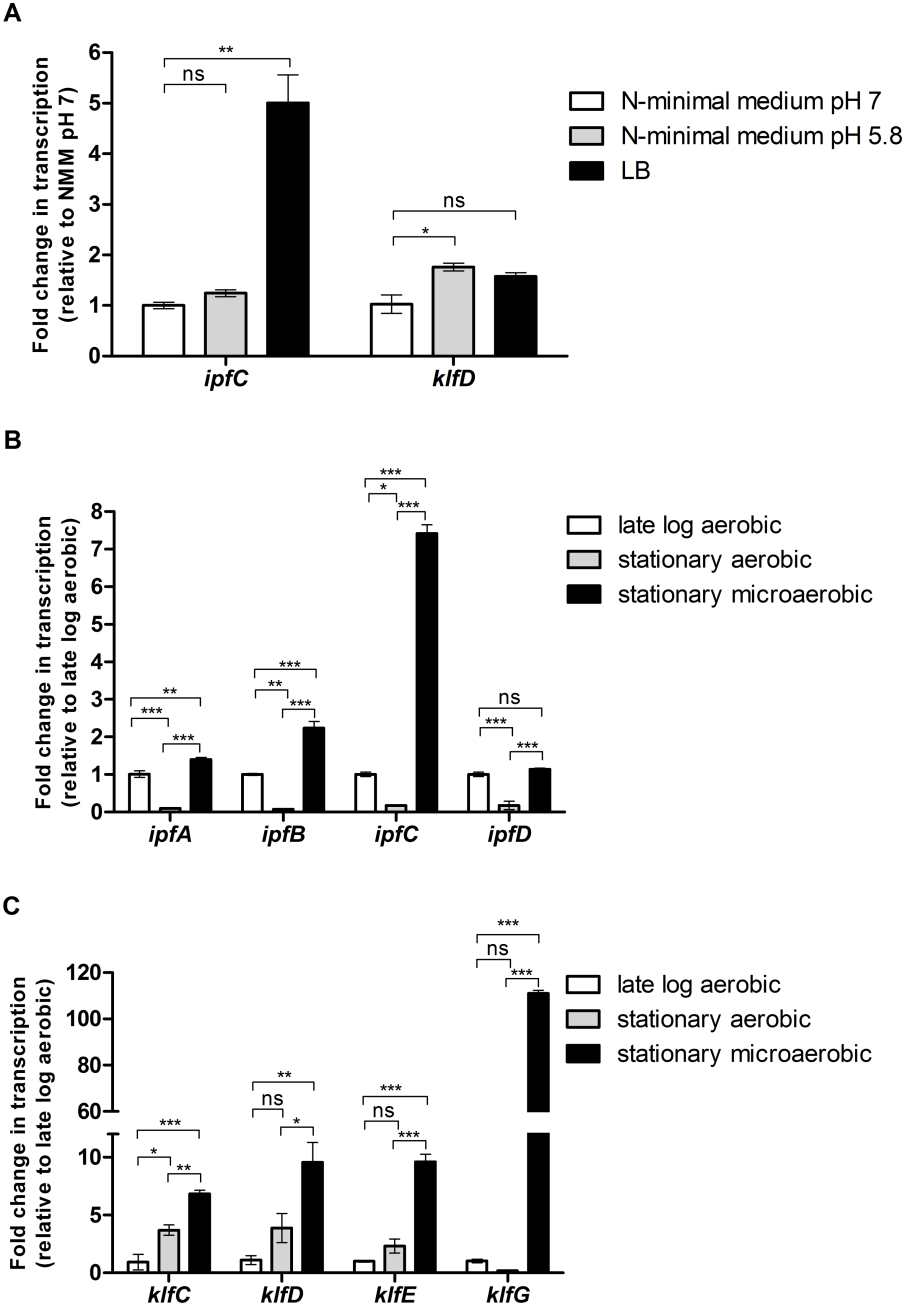
*klf* and *ipf* genes are induced under microaerobiosis. (**A**) RNA was extracted from *S*. Infantis wild-type strain grown to the stationary phase under microaerobic conditions in LB, N-minimal medium pH 7 and N-minimal medium pH 5.8. Quantitative RT-PCR analyses were conducted to determine the fold change in the transcription of *ipfC* and *klfD* in cultures grown in LB or N-minimal medium pH 5.8 relative to their transcription in N-minimal medium pH 7.0. (**B**) qRT-PCR analyses were performed to determine the fold change in the transcription of *ipfA*, *ipfB*, *ipfC* and *ipfD* in cultures grown in LB to the stationary phase under microaerobic conditions or under aerobic conditions, relative to their transcription at the late logarithmic phase under aerobic conditions. (**C**) qRT-PCR analyses were performed to determine the fold change in the transcription of *klfC*, *klfD*, *klfE* and *klfG* in cultures grown in LB to stationary phase under microaerobic or aerobic conditions, relative to their transcription at the late logarithmic phase under aerobic conditions. One way ANOVA with Dunnett's Multiple Comparison Test were performed to determine statistical significance. ns, not significant; *, P<0.05; **, P<0.01; ***, P<0.001.

Many of the chaperone-usher fimbriae genes identified in *E*. *coli* and *Salmonella* are poorly expressed under regular laboratory growth conditions [24,25] and were shown to be tightly regulated and assembled in response to specific environmental stimuli [26]. To study the native regulation of the Ipf and Klf fimbriae in *S*. Infantis we examined their expression under different growth conditions *in-vitro*. First, we determined their transcription during growth in rich LB broth, believed to mimic some of the intestinal environmental conditions [27] and in N-minimal medium pH 5.8 and 7.0, thought to mimic conditions found in the intracellular milieu [28]. Reverse-transcription real-time PCR (RT-PCR) analysis exhibited about 5-fold induction in the transcription of *ipfC* (encoding the Ipf usher) in cultures grown aerobically in rich LB broth relative to its transcription in minimal media (pH 7). In contrast, the transcription of *klfD* (encoding the Klf usher) was similar at rich and minimal media (Fig 5A).

Next we examined the transcription of Ipf and Klf fimbriae genes under aerobic vs. microaerobic conditions. All four Ipf genes (*ipfA*, *ipfB*, *ipfC* and *ipfD*) were found to be significantly induced under microaerobic conditions, when grown in LB to the stationary phase, compared to cultures grown to the stationary phase aerobically (Fig 5B). Similarly, all of the Klf genes tested (*klfC*, *klfD*, *klfE*, and *klfG*) were also found to be significantly induced, when grown to the stationary phase under the microaerobic growth conditions (Fig 5C), indicating that microaerobiosis is a potent stimulus for *klf* and *ipf* genes expression.

**Fig 6:**
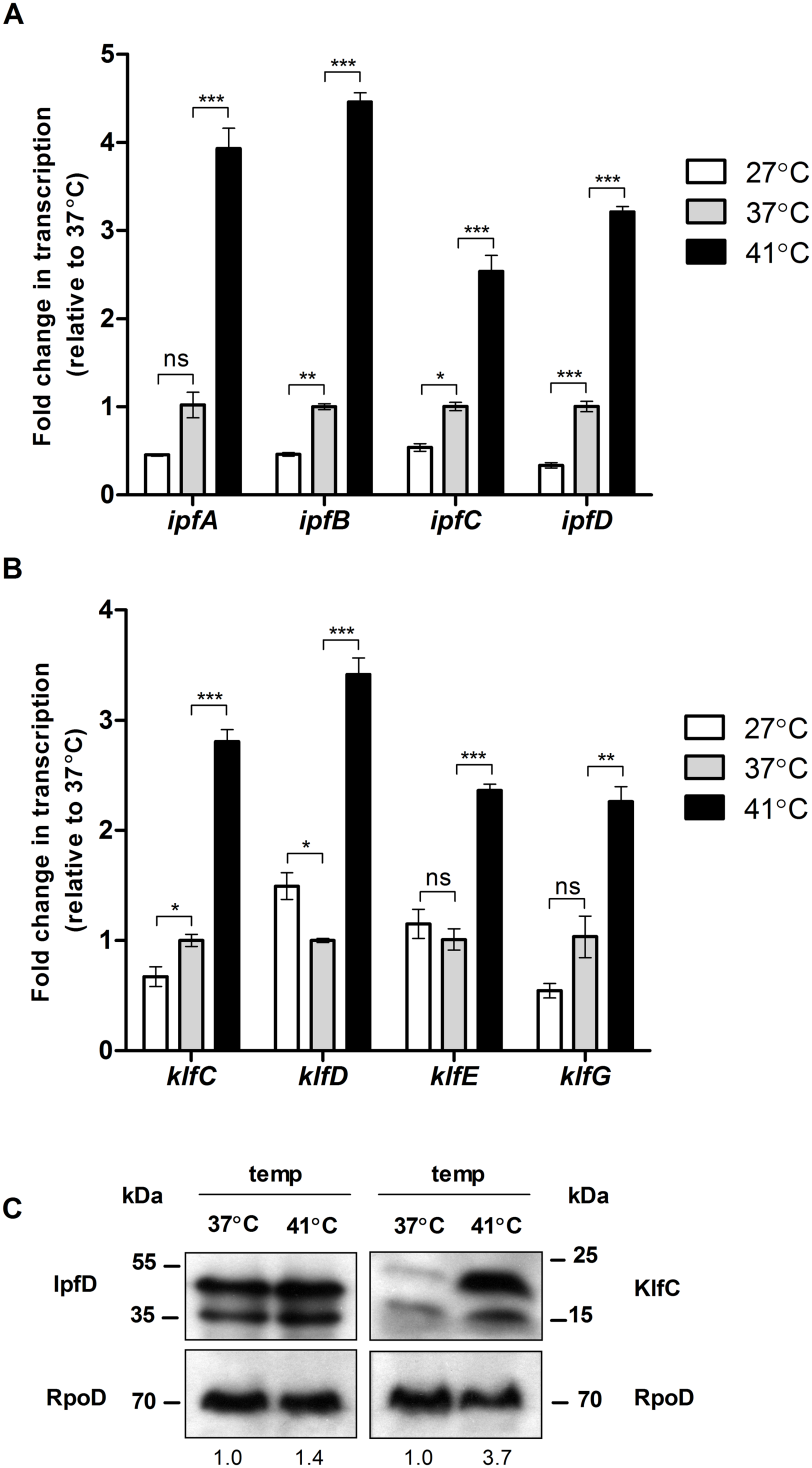
Klf and Ipf are induced at the avian physiological temperature. (**A**) qRT-PCR showing the fold change in the transcription of *ipfA*, *ipfB*, *ipfC* and *ipfD* in cultures grown in LB to the stationary phase under microaerobic conditions at 27°C or 41°C, relative to 37°C. (**B**) qRT-PCR showing the fold change in the transcription of *klfC*, *klfD*, *klfE* and *klfG* in cultures grown in LB to the stationary phase under microaerobic conditions at 27°C or 41°C, relative to 37°C. One way ANOVA with Dunnett's Multiple Comparison Test were performed to determine statistical significance. ns, not significant; *, P<0.05; **, P<0.01; ***, P<0.001. **(C)** Whole cell lysates of *S*. Infantis strains expressing a 2HA-tagged version of IpfD and KlfC that were grown in LB to the stationary phase under microaerobic conditions at 37°C and 41°C were separated on an SDS-PAGE. Western blotting using anti-HA antibody and anti-RpoD (as a loading control) are shown. The double band shown for KlfC-2HA and IpfD-2HA represents the premature and the signal peptide-cleaved forms of the proteins. IpfD-2HA and KlfC-2HA bands densitometry (normalized to the corresponding RpoD bands) are presented relative to the wt, under the RpoD blot.

Next, we investigated the expression of Ipf and Klf fimbriae during incubation at 27°C, 37°C and 41°C, corresponding to the temperatures of the environment, mammalian and avian hosts, respectively. Under growth in LB at 41°C, the transcription of *ipfA*, *ipfB*, *ipfC* and *ipfD* was upregulated by 4, 4.5, 2.5 and 3-fold respectively compared to the transcription at 37°C (Fig 6A). Similarly, the transcription of *klfC*, *klfD*, *klfE* and *klfG* was elevated by 2.5 to 3.5-fold under these growth conditions (Fig 6B). Consistent with these data, induction at 41°C was also demonstrated on the protein level as the expression of a 2HA-tagged version of IpfD was moderately higher at 41°C compared to 37°C. Even more so, considerably higher expression was exhibited for KlfC-2HA at 41°C compared to 37°C (Fig 6C). We concluded from these experiments that both Ipf and Klf fimbriae are induced at microaerobic conditions and under 41°C, a set of conditions found in the avian intestines.

### Lrp controls *klf* expression, while Fur and OmpR regulate *ipf*

Since the expression of both fimbriae was highly affected by microaerobic conditions, we sought to test a possible role of regulators known to be involved in oxygen homeostasis including OxyR, SoxR, ArcA, ArcB and FNR, [29]. In addition, we screened for potential regulatory roles of global regulators, previously reported to control *Salmonella* pathogenicity including RpoS, PhoP, OmpR and Fur [30] as well as the leucine-responsive regulatory protein (Lrp) that was shown to regulate different types of fimbriae in *E*. *coli* [31,32] and *Salmonella* [33].

**Fig 7:**
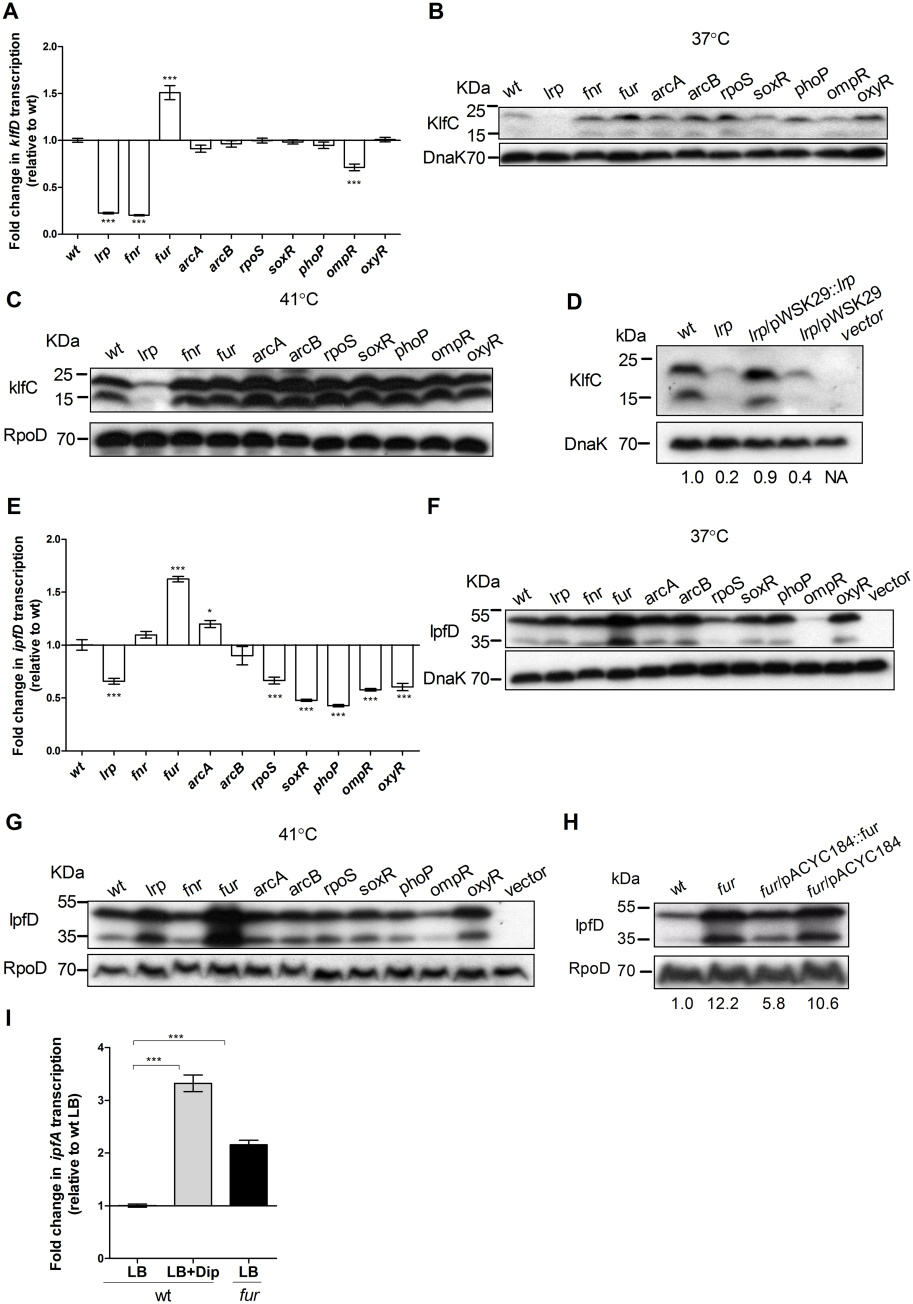
OmpR, Lrp and Fur are involved in pESI fimbriae regulation. **(A)** Total RNA was extracted from *S*. Infantis wild-type (wt) strain and ten isogenic null mutants harboring deletion in key regulatory genes. qRT-PCR was applied to define the fold change in *klfD* transcription between the wild-type background and the mutant strains. All cultures were grown in LB under microaerobic conditions at 37°C. **(B)** *S*. Infantis wild-type and ten derivative deletion mutant strains expressing KlfC-2HA were grown in LB under microaerobic conditions at 37°C. Bacterial lysates were separated by SDS-PAGE followed by western blotting using antibodies against hemagglutinin and DnaK as a loading control. **(C)** The same strains were grown in LB under microaerobic conditions at 41°C. Bacterial lysates were analyzed by western blotting using antibodies against hemagglutinin and RpoD. (**D**) *S*. Infantis wild-type strain (wt), its isogenic *lrp* null strain (*lrp*), an *lrp* mutant strain complemented with the *lrp* gene expressed from pWSK29 (*lrp*/ pWSK29::*lrp*), an *lrp* mutant harboring the empty vector (*lrp*/ pWSK29), all expressing KlfC-2HA from a low copy-number vector (pACYC184) were grown in LB broth under microaerobic conditions at 41°C. Wild-type strain harboring the empty plasmid pACYC184 (vector) was also included as a negative control. Bacterial lysates were separated by SDS-PAGE followed by western blotting using antibodies against hemagglutinin and RpoD. KlfC-2HA bands densitometry (normalized to the corresponding DnaK bands) are presented relative to the wt, under the DnaK blot. (**E**) qRT-PCR was applied to determine the fold change in *ipfD* transcription between the wild-type background and the mutant strains as in panel (A). (**F**) *S*. Infantis wild-type and ten isogenic mutant strains expressing IpfD-2HA were grown in LB under microaerobic conditions at 37°C. *S*. Infantis wild-type strain harboring the pWSK29 (vector) was used as a control for the western blotting. Bacterial lysates were separated by SDS-PAGE followed by western blotting as in panel (B). (**G**) The same strains were grown in LB under microaerobic conditions at 41°C. Bacterial lysates were analyzed by western blotting using antibodies against hemagglutinin and RpoD. (**H**) *S*. Infantis wild-type strain (wt), its isogenic *fur* mutant strain (*fur*), a *fur* mutant strain complemented with the *fur* gene expressed from pACYC184 (*fur*/ pACYC184::*fur*), and a *fur* mutant harboring the empty vector (*fur*/ pACYC184), all expressing IpfD-2HA from a low copy-number vector (pWSK29) were grown in LB broth under microaerobic conditions at 37°C. Bacterial lysates were separated by SDS-PAGE followed by western blotting using antibodies against hemagglutinin and RpoD. IpfD-2HA bands densitometry (normalized to the corresponding RpoD bands) are presented relative to the wt below the RpoD blot. (**I**) *S*. Infantis wild-type (wt) strain and its isogenic *fur* null mutant were grown at 37°C to the stationary phase under microaerobic conditions in LB or in LB supplemented with Dip (to a final concentration of 0.2 mM). qRT-PCR was applied to determine the fold change in *ipfA* transcription of the different cultures, relative to the wt strain grown in LB.

To study *klf* expression, qRT-PCR was applied to determine the fold change in *klfD* transcription in the *S*. Infantis wild-type strain in comparison to ten relevant isogenic regulatory mutants. This analysis showed more than 5-fold decrease in *klfD* transcription in the absence of Fnr and Lrp when cultures were grown at 37°C to the stationary phase (Fig 7A). Furthermore, sequence analysis of the *klfB* (the first gene in the operon) promoter region identified two putative Fnr binding sites (**TTGATTAAGATC**TG and C**TGATGCAGA**G**CA**G), similar to the known Fnr box consensus determined in *E*. *coli* (TTGATNNNNATCAA) [34], where N is any nucleotide and identical positions in the *klfB* promoter are highlighted in bold. Additionally, this region also includes 12 putative Lrp binding sites, all containing the consensus sequence GN(2–3)TTT recognized by Lrp [35] (S1A Fig), suggesting direct binding of Fnr and Lrp to this region.

To further characterize the potential role of the above regulators on the protein level, western blotting was used to determine the expression of a 2HA tagged version of KlfC in the wild-type relative to its expression in the above regulatory mutants at 37°C and 41°C. Although the expression of KlfC-2HA was significantly higher at 41°C than at 37°C, western blot analysis clearly demonstrated that the lack of LrP results in considerably lower amount of KlfC-2HA at both temperatures (Fig 7B and 7C). Moreover, ectopic expression of *lrp* from a low copy-number plasmid in the *lrp* background, but not the presence of the empty vector (pWSK29), complemented the KlfC-2HA expression to similar levels as in the wild-type (Fig 7D). In contrast to Lrp, we could not confirm any effect of Fnr on the expression of KlfC-2HA.

Similar approaches were also taken to study *ipf* regulation. qRT-PCR analysis that was applied for *ipfD* exhibited a negative regulatory role for Fur and showed moderately (two-fold or less) lower transcription of *ipfD* in the absence of Lrp, RpoS, SoxR, PhoP, OmpR, and OxyR compared to the wild-type background (Fig 7E). To confirm *ipfD* regulation on the protein level, the expression of IpfD-2HA in these mutant strains was further tested. While the potential role of some of these regulators was not confirmed by western blotting, this analysis clearly demonstrated, that IpfD-2HA is negatively regulated by Fur and that OmpR acts as a positive regulator of *ipfD* at 37°C (Fig 7F) and 41°C (Fig 7G). Moreover, expression of Fur from a plasmid that was introduced into the *fur* background, resulted in significantly reduced IpfD-2HA expression relative to its expression in the *fur* strain (Fig 7H).

Fur repression is known to occur due to the binding of the Fur-Fe^2+^ complex to a 19-bp consensus site (GATAATGATAATCATTATC) found in the operator of a target promoters [36]. Thus, to further confirm Ipf regulation by Fur, we studied the expression of *ipfA* in the presence of a Fe^2+^chelator, Dip (2,2'-dipyridyl). As shown in Fig 7I, adding Fe^2+^ chelating agent to the growth media resulted in more than three-fold induction of *ipfA* transcription, suggesting that Ipf expression is suppressed by Fe^2+^, likely via the Fur-Fe^2+^ complex. Consistent with these results *in-silico ipfA* promoter analysis identified putative imperfect 19-bp Fur binding site (T**A**A**AATG**G**TAATCA**AATGA) partially overlap with the -10 and the -35 sites in the *ipfA* promoter (S1B Fig). This observation provides further support to the possibility that Fur directly regulates *ipf* genes expression.

Collectively, we concluded from the above set of experiments that Lrp is an activator of the *klf* genes, and that the *ipf* operon is repressed by Fur and positively regulated by OmpR.

### KlfL and KlfB are negative and positive regulators, respectively of the *klf* cluster

**Fig 8:**
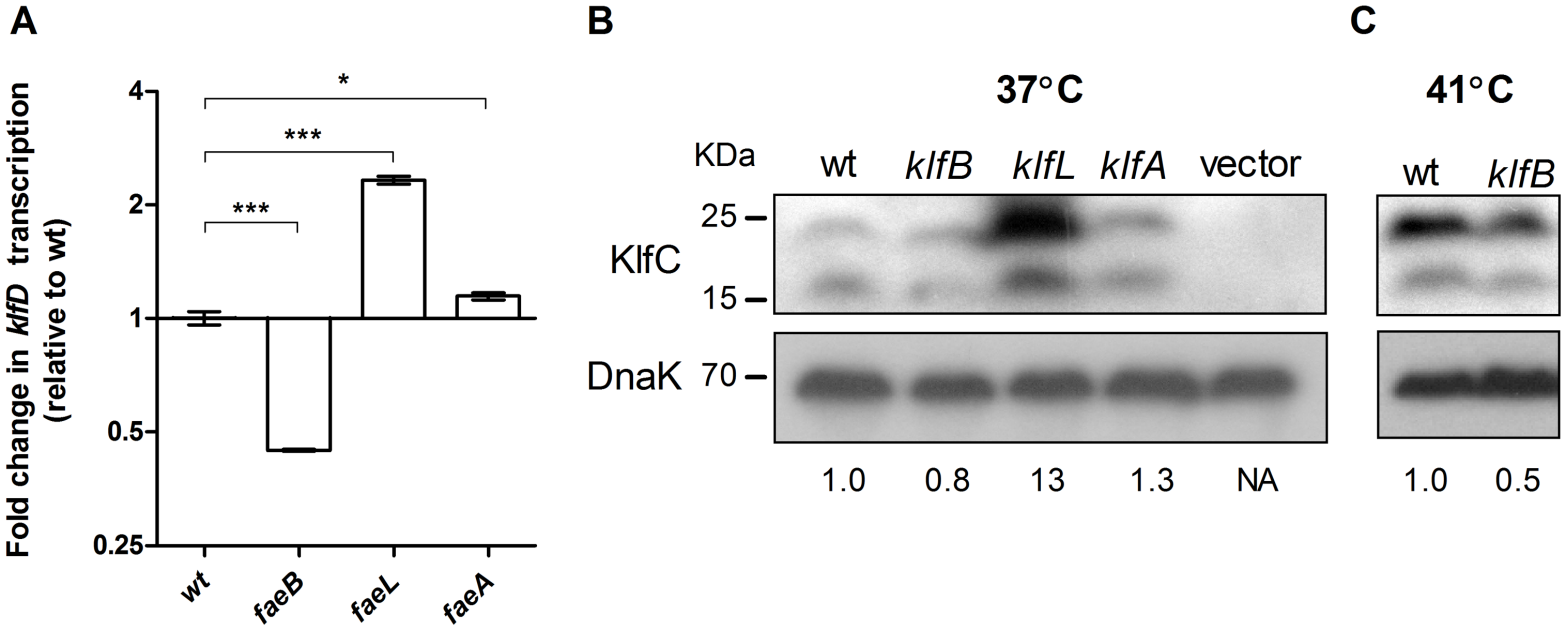
KlfB and KlfL are positive and negative regulators, respectively of the *klf* operon. **(A)** RNA was extracted from *S*. Infantis wild-type (wt) strain and three isogenic mutants containing null mutations in *klfA*, *klfB* and *klfL* grown in LB under microaerobic conditions at 37°C. qRT-PCR was conducted to define the fold change in *klfD* transcription between the wild-type background and the mutant strains. (**B**) *S*. Infantis wild-type and the *klfA*, *klfB* and *klfL* mutant strains expressing KlfC-2HA were grown to the stationary phase in LB under microaerobic conditions at 37°C. Wild-type strain harboring the empty plasmid pACYC184 (vector) was included as a negative control. Bacterial lysates were separated by SDS-PAGE followed by western blotting using antibodies against hemagglutinin and DnaK. KlfC-2HA bands densitometry (normalized to the corresponding DnaK bands) are presented relative to the wt, below the DnaK blot. (**C**) *S*. Infantis wild-type and the *klfB* mutant strain expressing KlfC-2HA were grown to the stationary phase in LB under microaerobic conditions at 41°C. Expression of KlfC-2HA was determined as in (B).

The presence of the unique region at the 5'-end of the *klf* cluster encoding *klfL* and *klfB* was intriguing, since it was not found in any other *klf*–related cluster in the database. BlastP analysis of KlfL showed a weak similarity to a *Mycobacterium* transcriptional regulator (WP_068209468) and KlfB was identical to a known regulator of the afimbrial adhesin AFA-III found in *E*. *coli* (WP_033555783) and *Salmonella* (WP_031619575). In addition, KlfA, located at the 3'-end of the *klf* cluster, also presented some homology (E-value 6e-14) to an uncharacterized *S*. *enterica* regulator (WP_061433569.1). Thus, the possible regulatory role of KlfL, KlfB and KlfA was studied next. In-frame null deletions were constructed for the three corresponding genes and the transcription of *klfD* was determined in these backgrounds compared to the *S*. Infantis wild-type strain. qRT-PCR showed more than twofold increase and twofold decrease in the transcription of *klfD* in the absence of *klfL* and *klfB*, respectively (Fig 8A).

These results were also confirmed on the protein level, where the amount of KlfC-2HA moderately decreased in the absence of *klfB*, but increased in the *klfL* backgrounds at 37°C (Fig 7B). Lower expression of KlfC-2HA in the *klfB* background was even more evident at 41°C (Fig 8C). We concluded from these analyses that KlfB and KlfL are positive and negative transcriptional regulators of the *klf* genes and that together with the core protein Lrp, are involved in the regulatory network, concerting the expression of the Klf fimbria.

### Differential expression of the *klf* and *ipf* operons in the mouse and chicken hosts

**Fig 9:**
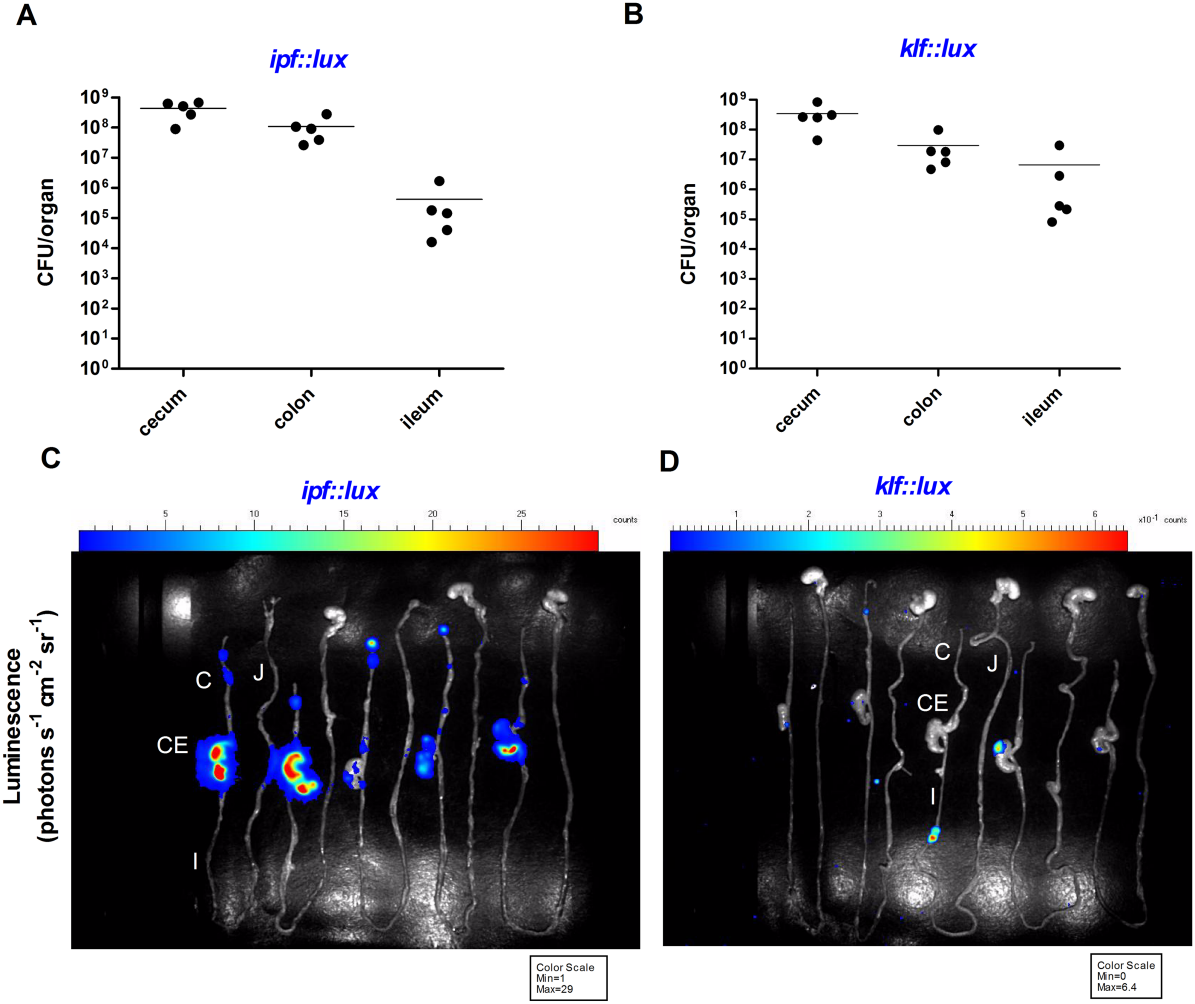
The expression profile of *ipf* and *klf* in the mouse model. Female C57BL/6 mice (five animals per group) were infected orally with ∼8×10^8^ CFU of *S*. Infantis strains harboring luciferase reporter fusion with *ipf (ipf*::*lux)* **(A, C)** and *klf* (*klf*::*lux*) (**B, D**). Twenty four hours p.i. mice were sacrificed and their intact GI tract was removed and imaged immediately using a photon-counting *in-vivo* imaging system. (**A** and **B**) Bacterial loads in the cecum, colon and ileum are indicated by total CFU counted in the entire organ. Each dot indicates the count in an entire organ in a single animal. (**C** and **D**) The detected bioluminescence signal is shown as pseudocolor images, with variations in color representing light intensity at a given location. The color bar indicates relative signal intensity (as photons s^−1^ cm^−2^ sr^−1^) and the minimal and maximal values measured are indicated in the box below each image. Different organs are indicated as follow: jejunum (J); ileum (I); cecum (CE); and colon (C).

To study the expression pattern of the Ipf and Klf fimbriae *in-vivo*, reporter gene fusions between the regulatory regions upstream to *ipfA* and *klfB* with the *luxCDABE* operon were constructed. *ipf*::*lux* and *klf*::*lux* fusions were both cloned into pCS26 and introduced to the *S*. Infantis wild-type strain. These strains were used to infect streptomycin pretreated C57BL/6 mice and one day-old chicks by oral gavage. At one day post infection (p.i.), the mice and the chicks were sacrificed and their entire gastrointestinal tract was imaged for luciferase activity. As shown in Fig 9, both reporter strains colonized the mouse intestines to a similar extent (Fig 9A and 9B). Nonetheless, while *ipf*::*lux* expression was clearly observed in the cecum and the colon of the infected mice (Fig 9C), expression of *klf*::*lux* was hardly detected (Fig 9D), suggesting low expression of the Klf fimbria in the murine host.

**Fig 10:**
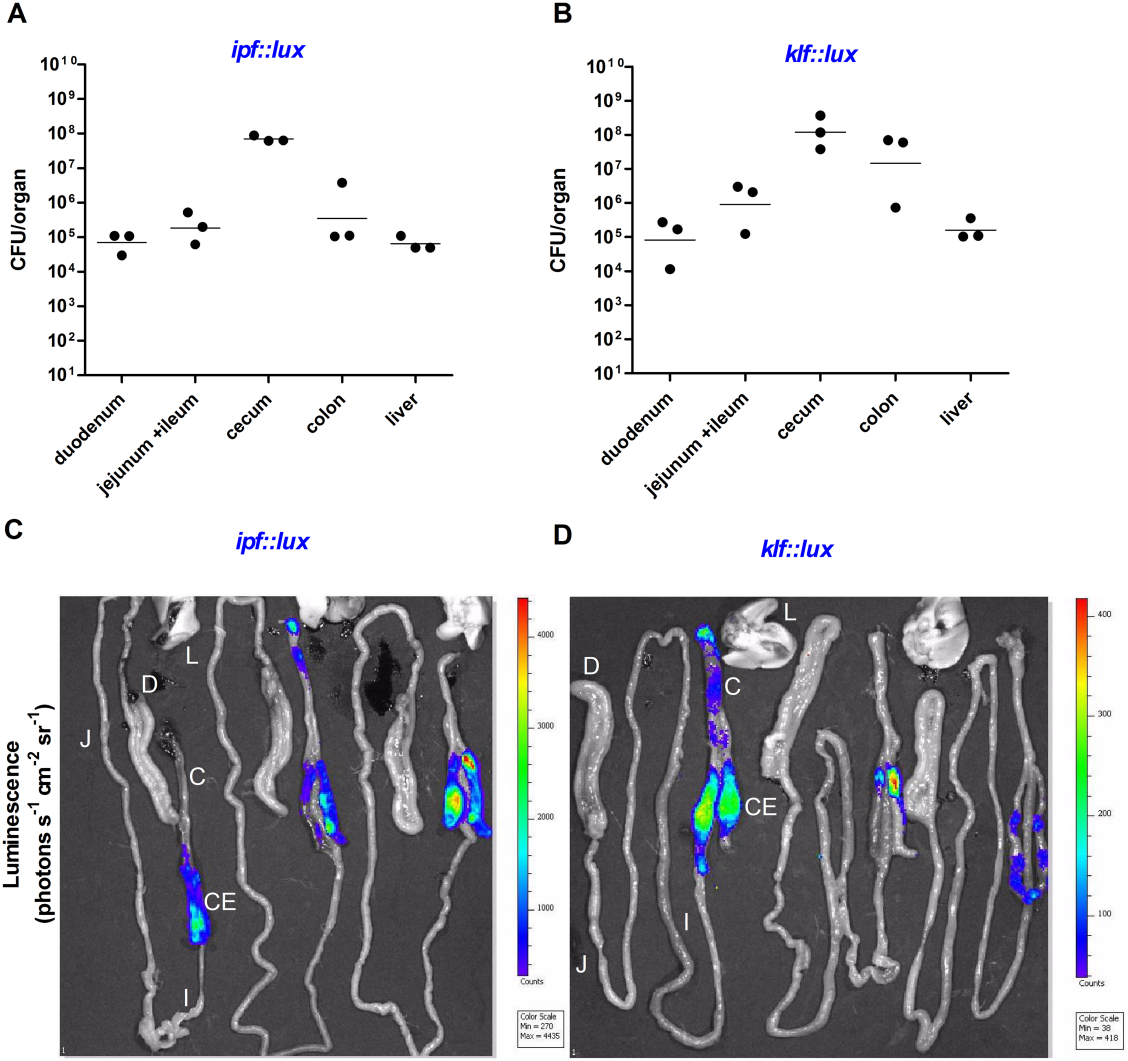
*klf* and *ipf* are significantly expressed in the cecum of infected chicks. Two groups of one day old White Leghorns chicks were infected orally with ∼1×10^7^ CFU of wild-type *S*. Infantis harboring the *ipf*::*lux* (**A, C**) and *klf*::*lux* (**B, D**) reporter strains. Twenty four hours p.i. chicks were sacrificed and their intact GI tracts as well as their liver were removed and imaged immediately using a photon-counting *in-vivo* imaging system. (**A** and **B)** Bacterial loads in the duodenum, jejunum and ileum, cecum, colon and liver is indicated by a CFU count per organ. The geometrical mean in each organ is shown by a solid horizontal line. **(C** and **D**) The color bar indicates relative signal intensity and the minimal and maximal values measured are shown in the box below the color bar. Different organs are designated as follow: duodenum (D) jejunum (J); ileum (I); cecum (CE); colon (C) and liver (L).

Intriguingly, in the chick model, a distinct expression pattern of these reporter genes was exhibited, compared to the one found in the mouse. First, while *klf*::*lux* was not expressed in the mouse intestines, it was noticeably expressed in the chick cecum and to a lower extent in the colon (Fig 10D). Secondly, although colonization in the chick and mouse cecum was similar (10^8^−10^9^ CFU), *ipf*::*lux* was expressed up to 150-fold higher in the chick cecum compared to the mouse (4435 vs. 29 photons S^-1^ cm^-2^ sr^-1^). Collectively, these results suggest that at day one p.i., both fimbriae are more induced in the chick model compared to the mouse. These results are in close agreement with the *in-vitro* data, showing induction of Klf and Ipf at 41°C vs. 37°C, corresponding to the body temperature of the avian and mammalian hosts, respectively.

### Ipf and Klf contribute differently to *S*. Infantis colonization in the mouse vs. the chicken hosts

To elucidate the contribution of the Klf and Ipf fimbriae to the *S*. Infantis virulence in the mouse and chicken hosts, competition experiments were conducted. In these infections, equal numbers of the wild-type and the mutant (*klf* or *ipf*) strains were used to co-infect streptomycin pretreated C57BL/6 mice and one day-old White Leghorns chicks. Four and three days p.i. the mouse and the chicks, respectively, were sacrificed and the bacterial load ratio between the fimbriae mutants and the wild-type strain (competitive index) was determined. Since competing strains were marked with different antibiotic cassettes, carried on the same backbone plasmid (pWSK29 for ampicillin and pWSK129 for kanamycin), a control group of mice was infected with the wild-type strains harboring pWSK29 or pWSK129. In the mouse model, competitive index was calculated for the ileum, cecum and colon since these are the main sites of *S*. Infantis colonization [18].

**Fig 11:**
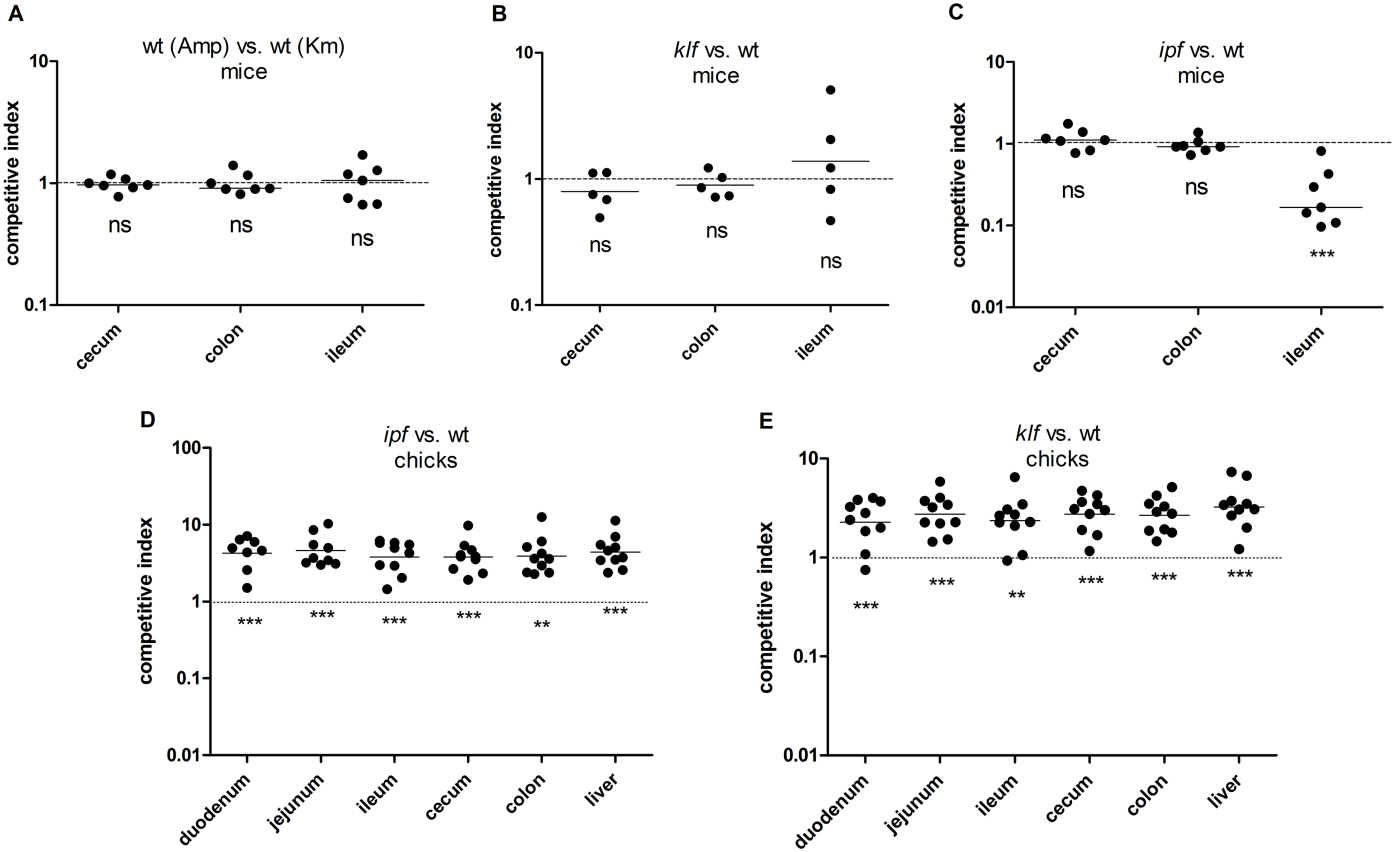
Ipf and Klf contribute differently to *S*. Infantis infection in mouse vs. chicken. (**A-C**) C57BL/6 mice were intragastrically infected with ~6×10^6^ CFU of a mixed (1:1) inoculum containing the wild-type *S*. Infantis (harboring pWSK129; Km^R^) and *klf* (**B**) or *ipf* (**C**) null mutant strain (harboring pWSK29; Amp^R^). A mixed inoculum of two *S*. Infantis wild-type strains carrying pWSK29 or pWSK129 was used as a control (**A**). Four days p.i., mice were sacrificed and tissues were harvested aseptically, homogenized and plated on selective XLD agar plates for bacterial enumeration. Each dot represents a competitive index (CI) value in one mouse in a single organ (cecum, colon or ileum). The CI was calculated as [mutant/wild-type]_output_/[mutant/wild-type]_input_. (**D** and **E**) Two groups of one day old SPF White Leghorns chicks were infected orally with ~1×10^7^ CFU of 1:1 mixed inoculum containing the wild-type *S*. Infantis (harboring pWSK129) and *ipf* or *klf* deletion mutant strains (harboring pWSK29). Three days p.i. chicks were sacrificed and the indicated organs were homogenized and plated on XLD agar plates supplemented with ampicillin or kanamycin for bacterial numeration. The CI was calculated as above.

Competitive infection with *S*. Infantis wild-type strains carrying pWSK29 (Amp) or pWSK129 (Km) showed equal colonization in the intestinal organs of the infected mice (Fig 11A), indicating equal fitness for both marked strains *in-vivo*. Competitive infection of the wild-type vs. the *klf* null strain showed no role for Klf in the pathogenicity of *S*. Infantis in the mouse, as equal bacterial loads of the wild-type and the *klf* mutant were recovered from the cecum, colon and ileum (Fig 11B). These results were not surprising, considering the very low expression of *klf*::*lux* in the mouse intestines. As for Ipf, while similar colonization was found for the wild-type and the *ipf* mutant strain in the cecum and colon, in the ileum, the *ipf* mutant was outcompeted by about 10-fold by the wild-type strain (Fig 11C). These results suggested that Ipf plays a role in *S*. Infantis colonization in the mouse ileum, possibly due to its capability to bind a yet unknown host receptor expressed at mouse ileum tissues, but not at the murine cecum or the colon.

Since we showed that the ambient temperature significantly affects the expression of the Ipf and Klf fimbriae, we were interested in characterizing their role in the chicken model, as its body temperature (41.2°C) is higher than the one of mice (~36.9°C). Surprisingly, when these competitive index infections were reproduced in the chick model, a different phenotype was observed. *ipf* outcompeted the *S*. Infantis wild-type strain by about 5-fold in the duodenum, jejunum, ileum, cecum, colon, and liver (Fig 11D). Similar results were also obtained for Klf, as the *klf* null strain outcompeted the wild-type in all of the tested organs by about 3-fold (Fig 11E), indicating moderately elevated colonization of the strains lacking the Klf or the Ipf fimbriae in comparison to the wild-type strain. To further examine this phenotype, we performed single infection experiment and infected three groups of one-day old chicks with 7×10^5^ CFU of *S*. Infantis wild-type, *klf* and *ipf* mutant strains. Five days p.i, the chicks were sacrificed and the bacterial load was determined in the cecum, ileum and colon. Consistent with the competition experiments, we found 4 to 83-fold higher numbers of the *klf* or the *ipf* mutant strains, than the wild-type *S*. Infantis background (S2 Fig); however, with seven chicks per group these differences did not reach statistical significance.

Accumulatively, we concluded that while the Klf fimbria is dispensable for *S*. Infantis colonization or that Ipf is required for maximal ileum colonization in the mouse, the expression of both fimbriae, may contribute to restrained bacterial burden of *S*. Infantis during chick infection, possibly due to their immunogenic nature and innate immune response to their induced expression by *S*. Infantis.

## Discussion

*Salmonella* Infantis is one of the clinically prevalent serovars worldwide and the most commonly reported serovar in broilers in Europe [37]. In the last two decades, emergence of resistant *S*. Infantis clones has been reported in many countries including USA [21], Belgium and France [38], Germany [39], Italy [20], Hungary [40], Japan [41], Honduras [42] and Israel [19]. Our previews work established that the clonal emergence of *S*. Infantis in Israel involved the acquisition of the pESI megaplasmid [18,43]. More recently, related plasmids were also found to be associated with the increased prevalence of *S*. Infantis clones in other countries [20,21,44], inferring that gaining these related megaplasmids may play an important role in the successful spread of *S*. Infantis.

Besides antibiotic resistant genes, dozens of other ORFs, most of which with unknown functions are encoded on these plasmids and at least some of these ORFs are expected to significantly alter *Salmonella* interaction with the host [18] and its microbiota [43]. Here we imaged and elucidated the genetic organization, regulation and role in virulence of two novel chaperone-usher fimbriae encoded within pESI and demonstrated how they affect *S*. Infantis colonization in the chicken vs. the mouse hosts.

Blast search against the *S*. Infantis genome indicates that the 119944 strain encodes at least twelve different chaperone-usher clusters, which is comparable with the number of chaperone-usher fimbriae harbored by other subspecies I serovars, containing on average twelve fimbrial gene clusters [10]. Nevertheless, the Ipf fimbria is rather unique and Ipf homologues were identified only in subsp. diarizonae and subsp. VII. Horizontal gene transfer of the *ipf* cluster from a distant origin may expand the ecological niches and host adaptation of *S*. Infantis carrying pESI.

In contrast to the scarce distribution of Ipf, homologous clusters of the Klf fimbria were found in at least seven subsp. I serovars. Yet, a detailed comparison showed that despite the high conservation of the Klf proteins, KlfG is highly diverse. The KlfG homolog in ETEC, FaeG was shown to form the major structural component of the K88 fimbria that also mediates the adhesive properties of the fibers [45,46]. Allelic diversity in the major adhesive subunit, is likely to provide different binding properties as was previously shown for K88 variants in ETEC [23] and for FimH [13,47]. The mechanisms by which genetic variation occurs only in KlfG and not in the other subunits of the Klf fimbria that are highly conserved are still unknown. Another feature unique to the pESI-*klf* cluster is the insertion of two ORFs (*klfL* and *klfB*) in the 5'-region of the cluster that were found to act as *klf* genes regulators. These observations demonstrate two mechanisms of fimbria evolution, occurring by: (i) modification of a structural adhesive subunit (KlfG) and (ii) subordinating the fimbrial expression under newly acquired regulators.

Besides KlfB and KlfL that were shown to act as positive and negative regulators, respectively, Lrp was established to positively regulate Klf expression. Lrp is a global regulator that can function as a repressor or activator of transcription, controlling the expression of numerous operons in *E*. *coli* and *Salmonella* [48]. In addition to operons involved in amino acid metabolism, Lrp was previously shown to regulate different fimbrial operons including *pap* (P pilus) and *fan* (K99) [31], *fim* [33,49], *sfa*, *daa* [50], as well as the nonfimbrial adhesin TosA in uropathogenic *E*. *coli* [32]. Interestingly, while Lrp was previously reported to negatively regulate the *fae* operon (by cooperative binding with FaeA) in *E*. *coli* [51], here we showed that in *S*. Infantis, Lrp acts as an activator, indicating substantial differences in the regulatory setup between the *fae* genes in ETEC and the *klf* genes in *S*. Infantis. Such differences may be due to the differences in the ecology or lifestyle of these pathogens and the necessity to coordinate *klf* expression with other *Salmonella* virulence regulons such as *Salmonella* pathogenicity island 1 [52].

Like Lrp, other core global regulators including, Fur and OmpR were found to control *ipf* expression. Negative regulation of fimbria by Fur has been previously reported for the CFA/I fimbriae of ETEC [53] and type 3 fimbriae in *Klebsiella pneumonia* [54]. In many bacteria, Fur is generally involved in regulation of iron homeostasis genes, but it is also known to participate in bacterial colonization, oxidative stress response, toxin secretion and pathogenicity [55]. Under iron-abundant conditions, Fur-Fe^2+^ dimers bind to the Fur box in target promoters, which interfere with the binding of RNA polymerase and thus preventing transcription from these genes [56]. The fact that *ipf* expression is upregulated in the presence of an iron chelator and the identification of putative Fur box in the promoter of *ipfA* strengthen the possibility that Ipf regulation by Fur is direct. Derepression of *ipf* in the absence of Fur-Fe^2+^ is expected to contribute to the induction of this fimbria when *S*. Infantis is translocated from the environment (or rich iron milieu) to the host's small intestine, thought to be scarce in free ferrous [57].

While the regulatory role of Lrp, Fur and OmpR was shown both on the RNA and the protein levels, the absence of other regulators such as Fnr, PhoP and SoxR was more evident transcriptionally, but not on the protein level. This may occur due to indirect activity of these pleiotropic regulators and additional posttranscriptional regulatory mechanisms involved in the expression of *klf* and *ipf* genes. Also, the presence of secondary promoters and internal regulatory elements within the fimbria clusters cannot be excluded and requires further investigation.

Besides iron availability, another environmental stimuli that were found to regulate the expression of both fimbria are microaerobiosis and elevated temperature of 41°C, characterizing the intestinal conditions of the avian hosts. Induction of fimbria expression under oxygen limitation was previously reported for the MR/P fimbria of uropathogenic *Proteus mirabilis* and type 1 fimbriae of uropathogenic *E*. *coli* [58]. Thermoregulation of fimbria expression has been shown for the BAV1965-1962 fimbrial locus of *Bordetella avium*, which was expressed at 37°C, but not at 22°C [59]. The 987P fimbriae of ETEC was also reported to be induced at 37°C and not when grown at lower temperatures [60]. In contrast, the expression of the F9 fimbriae in uropathogenic *E*. *coli* was shown to be repressed by H-NS at 37°C or 28°C and induced at 20°C, while mediating significant biofilm formation at the lower temperature [61]. Similarly, the *E*. *coli* Mat fimbria and curli fibers were also reported to be expressed strongly at 20°C [62,63]. These examples suggest that both temperature and oxygen concentration are used by different pathogens to sense the environment and regulate various fimbriae expression according to their location. Here we showed that both pESI-encoded fimbriae are induced *in-vitro* at 41°C compared to the ambient temperature (27°C) or 37°C, suggesting that these fimbriae might be expressed differently according to the type of the infected host. In agreement with this idea, we showed significantly higher induction of Ipf and Klf in chicks compared to mice, differentiated by physiological temperature of 41.2°C and 37°C, respectively.

Intriguingly, not only the expression profile was different between the chick and the mouse hosts, but also the contribution of these fimbriae to the infection varied between these hosts. While a *klf* mutant did not present any detectable phenotype in competitive infections in mice (likely due to the lack of Klf expression in this host), this mutant strain colonized moderately better the chick intestines than the wild-type. A different, or even an opposite effect on colonization was also found for Ipf. Whereas an *ipf* mutant strain showed colonization deficiency in the ileum of mice, in the chick model it outcompeted the wild-type strain by about 5-fold. Superior colonization of the *klf* and *ipf* mutants in chicks is likely to occur due to a stronger innate immune response against these fimbriae in the chick vs. the mouse, possibly as a result of their induced expression in the former host. Function of these surface exposed structures as pathogen associated molecular patterns (PAMPs) and their recognition by the avian innate immune system is expected to result in a better infection control of the wild-type compared to the fimbria mutant strains. Consistent with this notion, are recent studies reported that FimH, the adhesive component of the type 1 fimbriae, is a potent inducer of innate antimicrobial responses mediated by TLR4 and type 1 interferon signaling in mice, and that FimH induces significant levels of NO production [64,65]. Thus, our working model proposes that the higher body temperature in the avian host leads to increased expression of the Ipf and the Klf fimbriae, which induces a stronger immune response against *S*. Infantis infection. Efficient immune response will result in lower colonization of the fimbriae-positive strain in chicks. We further speculate that stronger immune response against strains that express these fimbriae may restrain the acute infection by *S*. Infantis and support persistence in the bird. These results may explain the increased prevalence of the pESI or pESI-like positive stains in poultry as was previously reported [19,20].

It is well established that different composition of the fimbriome contributes to host tropism and that different fimbriae or even allelic variation within a particular fimbria can change the interaction of *Salmonella* with its host [13,47,66]. Our study demonstrates additional mechanism affecting fimbriae diversity. We showed here that fimbriae expression can be altered between hosts, most likely, in response to their physiological temperature. We suggest that by coordinating fimbriae expression according to the host temperature, *Salmonella* can express a different set of fimbriae in mammalian vs. avian hosts and by that shape the outcome of the infection. While moderate expression of a particular fimbria may facilitate colonization (as was shown for Ipf in the mouse ileum), it is possible that higher expression may act as a two-edged sword that elicits stronger immune response. A robust immune response will restrain bacterial burden (as was shown for Ipf and Klf in chicks), in a way that may facilitate a persistent infection.

## Materials and methods

### Bacterial strains, media and growth conditions

Bacterial strains utilized in this study are listed in S4 Table. Bacterial cultures were routinely maintained in Luria-Bertani (LB; Lennox) broth (BD Difco) or in N-minimal medium containing 80 mM MES (for pH 5.8) or 100 mM Tris-HCl (for pH 7.0), 5 mM KCl, 7.5 mM (NH_4_)SO_4_, 0.5 mM K_2_SO_4_, 337 μM K_2_HPO_4_/KH_2_PO_4_, 20 mM MgCl_2_, 38 mM glycerol, and 0.1% Casamino acids as indicated and were plated onto LB or xylose lysine deoxycholate (XLD; BD Difco) agar plates. To chelate Fe^2+^, 2,2'-dipyridyl (Dip; Sigma-Aldrich) was added to LB at final concentration of 0.2 mM. Aerobic cultures were inoculated in 2 ml medium and grown in 15 ml glass tubes with vigorous shaking (250 RPM). Microaerobic cultures were grown by diluting 1:100 over-day aerobic culture into 10 ml medium transferred into 15 ml tubes that were incubated for 16 h without shacking, with the lid loosely screwed in. When appropriate, antibiotics were added to the medium as follows: tetracycline (20 μg/ml), kanamycin (50 μg/ml), ampicillin (100 μg/ml) and chloramphenicol (25 μg /ml).

### Molecular biology and cloning

All primers used in this study are listed in S5 Table. Oligonucleotides were purchased from IDT and PCR was carried out using Phusion Hot Start Flex DNA Polymerase (New England BioLabs) or with ReddyMix PCR (Thermo Scientific). All *S*. Infantis null mutants were constructed using the λ-red-recombination system and a three step PCR method to produce an amplimer containing the antibiotic resistance gene, as described in [67]. Resistant cassette was then eliminated from the genome by using a helper plasmid encoding the FLP recombinase [68]. For western blotting, a C-terminal two-hemagglutinin (2HA) tagged version of KlfC from *S*. Infantis was cloned into pACYC184 cut with *Sal*I and *Bgl*II and a 2HA C-terminal IpfD tagged was cloned into pWSK29 using *Sac*I and *Xba*I. For expression of *ipf* and *klf* under arabinose inducible promoter, a PCR fragment containing the *ipfABCD* was obtained using the primers '*ipf* pBAD Fw' and ' *ipf* pBAD Rv' and digested with *Sac*I and *Xba*I. A PCR fragment containing *klf*BCDEFGHIJKA was obtained using the primers *' fae*-k88 pBAD Fv' and *' fae*-k88 pBAD Rv' digested with *Nhe*I and *Sac*I. Both fragments were then cloned into pBAD18. For *fur* and *lrp* complementation, the intact sequence of the two regulators was PCR amplified using *S*. Infantis 119944 as a template including their native regulatory regions containing 257 and 427 nucleotides upstream from the first methionine, respectively. *fur* was amplified using primers 'fur *Sal*I Fw' and 'fur *Hind*III Rv', digested with *Sal*I and *Hind*III and cloned into pACYC184. *lrp* was amplified using primers 'lrP *Sac*I Fw' and 'lrp *Xba*I Rv', digested with *Sac*I and *Xba*I and cloned into pWSK29. The pWSK29::*lrp* and pACYC184::*fur* were transformed into *S*. Infantis *lrp* and *fur* null mutant strains, respectively. For *in-vivo* imaging of *ipf* and *klf* expression, the regulatory region containing 421 and 396 nucleotides upstream from the first methionine of IpfA and KlfB, respectively was amplified with the primers '*ipf* promoter *Xho*I Fw' and '*ipf* promoter *Bam*HI Rv', and 'K88 promoter *Xho*I Fw' and 'K88 promoter *Bam*HI Rv', respectively, digested with *Bam*HI and *Xho*I, and cloned into pCS26.

### Reverse transcription real-time PCR

RNA was extracted from *S*. Infantis cultures grown under different conditions using the Qiagen RNA protect bacterial reagent and the RNeasy mini kit (Qiagen) according to the manufacturer's instructions, including an on-column DNase I digest. Purified RNA was retreated with an RNase-free DNase I followed by ethanol precipitation. 200 ng of DNase I-treated RNA was subjected to cDNA synthesis using the iScript cDNA synthesis kit (Bio-Rad Laboratories). Real-time PCR and data analysis were performed as previously described [69] on a StepOnePlus Real-Time PCR System (Applied Biosystems). The 16S rRNA gene was used as the endogenous normalization controls. Fold-differences in gene transcription were calculated as 2^-ΔΔ^C_t_.

### Western blotting

*Salmonella* cultures were grown in LB to the stationary phase under microaerobic conditions at 37°C or 41°C, as indicated. The cultures were OD_600_ normalized, centrifuged, and pellets were resuspended in 1× sodium dodecyl sulfate-polyacrylamide gel electrophoresis (SDS-PAGE) sample buffer. Boiled samples were separated on 10% or 12% SDS-PAGE and transferred to a polyvinylidene fluoride (PVDF) membrane (Bio-Rad Laboratories). Blots were probed with anti-HA tag antibody (Abcam; ab18181, diluted 1:1,000); anti-RpoD antibody (Santa Cruz Biotechnology; SC56768, diluted 1:2,000) or anti-DnaK (Abcam; ab69617, diluted 1:10,000), when the marketing of the anti-RpoD antibody was discontinued by the manufacturer. Goat anti-mouse antibody conjugated to horseradish peroxidase (Abcam; ab6721, diluted 1:5,000) was used as a secondary antibody, followed by detection with enhanced chemiluminescence (ECL) reagents (Amersham Pharmacia).

### *In-vivo* imaging during murine and poultry infection

The regulatory regions of *ipf* and *klf* were cloned into pCS26 upstream to a promoterless *luxCDABE* operon (pCS26::P*ipf* and pCS26::P*klf*, respectively) and transformed into *S*. Infantis 119944. The reporter strains were grown for 16 h in LB supplemented with kanamycin at 37°C and ~8×10^8^ CFU were used to infect by oral gavage female C57BL/6 mice (Envigo, Israel) that were pretreated with streptomycin. Similarly, one day old SPF White Leghorns chicks (Charles River) were infected orally (inter crop) with ~1×10^7^ CFU of the reporter strains in 0.2 ml saline. At 24h p.i., mice and chicks were sacrificed and their intact gastrointestinal tracts were removed and imaged using a photon-counting *in-vivo* imaging system (Photon-Imager, Biospace Lab or IVIS Lumina LT, PerkinElmer). To determine bacterial loads, the organs were homogenized in 0.7 ml saline using a BeadBlaster 24 homogenizer (Benchmark Scientific), serially diluted and plated on XLD agar plates supplemented with kanamycin.

### Murine and poultry competitive index (CI) infections

Eight to ten week old female C57BL/6 mice (Envigo, Israel) were pretreated with streptomycin (20 mg per mouse in 100 μl HEPES buffer) 24 h prior to infection. Mice were infected with ~6×10^6^ CFU of a mixed (1:1) inoculum containing the wild-type *S*. Infantis (harboring pWSK129; Km^R^) and *ipf* or *klf* null mutant strain (harboring pWSK29; Amp^R^). A mixed inoculum of two *S*. Infantis wild-type strains carrying pWSK29 or pWSK129 was used as a control.

SPF eggs of White Leghorns chicks (Charles River) were incubated for 21 days at 37.4°C in SPF chicken isolators. One day after hatching, the chicks were orally (inter crop) infected with ~1×10^7^ CFU of 1:1 mixed inoculum containing the wild-type *S*. Infantis (harboring pWSK129) and *ipf* or *klf* null mutant strain (harboring pWSK29) in 0.2 ml saline. The bacteria strains for both hosts were grown aerobically with the appropriate antibiotics for 16 h in LB at 37°C. Four and three days p.i. mice and chicks, respectively were sacrificed and the GI organs (including the intestinal contents) were collected on ice and homogenized in 0.7 ml saline. Serial dilutions of the homogenates were plated on XLD agar plates supplemented with ampicillin or kanamycin. CFUs were counted and the competitive index was calculated as [mutant/wild-type]_output_/[mutant/wild-type]_input_.

### Heterologous expression of the fimbria and mass spectrometry

A non-fimbriated *E*. *coli* (ORN172) strains carrying the *ipf* operon (pBAD18::*ipf*), the *klf* operon (pBAD18::*klf*) or the empty vector (pBAD18) were grown aerobically overnight in LB supplemented with ampicillin at 37°C. The next day, the cultures were washed twice with N-minimal medium and diluted 1:50 into N-minimal medium pH 7 containing ampicillin (100 μg/ml), L-arabinose (50 mM) or glucose (1M) and grown for 6 h until reaching OD_600_ of 1. OD_600_-normalized cultures were centrifuged and resuspended in 2 ml phosphate-buffered saline (PBS). Surface exposed fimbriae were separated from the cells by mechanical shearing using a shaft blender (three cycles of 1 min each). Cellular debris was removed by centrifugation (13,000 rpm, 5 min at 4°C), the supernatant was collected and filtered using a 0.22-μm filter (Merck Millipore). The filtered supernatant was then precipitated in 10% Trichloroacetic acid (TCA) for overnight on ice. Precipitated fractions were recovered by centrifugation (13,000 rpm, 45 min at 4°C) and the pellet was washed with 0.8 ml of ice-cold acetone. After acetone was removed, the pellet was air-dried for 10 min at room temperature in a fume hood and resuspended in 20 μl of 1× SDS-PAGE sample buffer. The boiled samples were separated on 12% SDS-PAGE followed by Coomassie Blue staining. Bands that were unique or significantly enriched relative to the negative control (pBAD18) were cut from the gel and were subjected to mass spectrometry analysis at the Smoler Proteomic Center at the Technion, Haifa, Israel. The samples were digested by trypsin, analyzed by LC-MS/MS on LTQ-Orbitrap (Thermo) and identified by Discoverer 1.4 software using two algorithms: Sequest (Thermo) and Mascot (Matrix science) against the fimbrial subunits sequences and the *E*. *coli* proteome from the Uniprot database and a decoy database, in order to determine the false discovery rate (FDR). High confidence peptides have passed the 1% FDR threshold.

### Atomic force microscope (AFM) and transmission electron microscopy (TEM)

The *ipfABCD* operon was amplified by PCR using primers 1f-SIN-ipf and 1r-SIN-ipf. The product was cloned under the control of a tetracycline-inducible *tetR* P_*tetA*_ promoter element on plasmid vector pWSK29 by Gibson assembly according to manufacturer´s protocol (NEB). Detailed information about the cloning strategy is given in Hansmeier *et al*. (2017, in revision). The plasmid was introduced into *E*. *coli* ORN172 strain and the culture was grown in LB supplemented with 50 μg/ml carbenicillin for overnight. To induce the fimbriae expression, an overnight culture was subcultured (1:31) in fresh LB medium supplemented with 50 μg/ml carbenicillin and 100 ng/ml anhydrotetracycline (AHT) and grown for 3.5 h. 150 μl of the subculture were applied on a cover-slip of 12 mm diameter, allowed to dry at air for 1 h, and washed four times with ddH_2_O before imaged by AFM. Measurements were performed under ambient conditions using the NanoWizard II AFM system (JPK Instruments AG) in the soft contact mode using silicon nitride AFM probes with a constant nominal force of 0.06 N/m (SiNi, Budget Sensors, Wetzlar). Scan rates were set to 1 Hz and images were recorded at a resolution of 512 x 512 pixel. Representative height and deflection images are shown in false color. Using the JPK data processing software (JPK Instruments AG) all images were tilt corrected, polynomial fitted and unsharpened mask filtered. Images were adjusted for brightness and contrast using PhotoShop (Adobe). Height measurements of Ipf fimbriae were made with the JPK data processing software of bacteria (n = 18) from three individual replicates. For TEM imaging, 500 μl of the subculture grown as described before for heterologous expression analyses were pelleted for 5 min at 1,000 x g. The pellets were carefully resuspended in 500 μl ddH_2_O. 3 μl of bacterial suspension was dropped on formvar/carbo-coated TEM grids that were glow-discharged using the plasma cleaner (Diener electronic) shortly before preparation. Suspensions were left for 1 min to allow absorption of bacteria. Residual suspension was removed with a filter paper. Grids were negatively stained with 0.5% phosphotungstic acid, pH-adjusted to 7.4, blotted with filter paper and finally air dried. TEM analysis was performed using a Zeiss 902 system operating at 50 kV.

### Bioinformatics tools

The pESI-encoded *ipf* and *klf* gene clusters were compared using the Easyfig tool (http://mjsull.github.io/Easyfig/) with homologous clusters found in the nr database at NCBI. Promoter location including the -10 and -35 boxes was predicted by BPROM (http://www.softberry.com/berry.phtml?topic=bprom&group=programs&subgroup=gfindb). The Fnr, and Fur binding sites were predicted by the Virtual Footprint tool (http://www.prodoric.de/vfp/vfp_promoter.php). Multiple sequence alignment of the KlfG homologs was performed using CLUSTALW (http://www.ch.embnet.org/software/ClustalW.html) and BOXSHADE 3.2 (http://www.ch.embnet.org/software/BOX_form.html) tools. The signal peptide sequence preceding the Sec cleavage site in KlfG was predicted by the SignalP 4.1 program (http://www.cbs.dtu.dk/services/SignalP/).

### Statistics

Statistical analysis was performed using the GraphPad Prism 5 software package (GraphPad Software, Inc,). Analysis of variance (ANOVA) with Dunnett's multiple comparison test was used to determine differences between multiple data sets. A student t-Test against a theoretical mean of 1.0 was used to determine statistical significance of the C.I values. *P*-value smaller than 0.05 was considered statistically significant and was indicated in the figures as follow: *, *P* <0.05; **, *P* <0.01; ***, *P* <0.001; ns, not significant. Error bars show the standard error of the mean.

### Ethics

Mice and chicks experiments were conducted according to the ethical requirements of the Animal Care Committee of the Sheba Medical Center (Approval numbers 933/14 and 1059/16, respectively) and in line with the guidelines of the National Council for Animal Experimentation.

## Supporting information

S1 Table Sequence similarity between the *S*. Infantis Ipf proteins and homologous proteins in other *Salmonella* serovars. (PDF)

S2 Table Homology of the Klf subunits to *E*. *coli* K88-Fae fimbria. (PDF)

S3 Table Sequence similarity between the *S*. Infantis Klf proteins and other *Salmonella* serovars. (PDF)

S4 Table Bacterial strains utilized in this study. (PDF)

S5 Table Primers used in this study. (PDF)

S1 Fig *ipfA* and *klfB* promoter analysis. DNA sequences containg 574 bp upstream from *klfB* and *ipfA* were analysed *in-silico*. Promoter location including the -10 and -35 boxes was predicted by BPROM, and the Fnr and Fur binding sites were predicted by the Virtual Footprint tool. (**A**) The *klfB* promoter region. -10 and -35 sites are highlighted in green and indicated by red text, predicted Fnr binding sites are in bold font and highlighted in yellow. Twelve putative Lrp binding sites (Lrp1-12) are numbered and underlined. (**B**) The *ipfA* promoter region. -10 and -35 sites are highlighted in green and the predicted Fur binding site is in bold font and underlined.
(TIF)

S2 Fig Three groups of one day old SPF White Leghorns chicks (seven birds per group) were infected orally with 7×10^5^ CFU of *S*. Infantis wild-type background and its isogenic *ipf* and *klf* null mutant strains. Five days p.i. chicks were sacrificed and the indicated organs were homogenized and plated on XLD agar plates supplemented with tetracycline for bacterial numeration. Bacterial burden in the cecum, ileum and colon is shown. Each dot represents the count in a single entire organ. The geometrical mean in each organ is shown by a solid horizontal line.
(TIF)
