# Supplementary material for: The plasmid-encoded Ipf and Klf fimbriae display different expression and varying roles in the virulence of Salmonella enterica serovar Infantis in mouse vs. avian hosts
Source: PLoS Pathog. 2017 Aug 17;13(8):e1006559. doi: 10.1371/journal.ppat.1006559 (PMC5560535; doi:10.1371/journal.ppat.1006559)
Supplement: S1 Table — (PDF) [file ppat.1006559.s001.pdf]

S1 Table. Homology[*] between the *S.* Infantis Ipf proteins and other *Salmonella* serovars

S. Infantis pESI	IpfA	IpfB	IpfC	IpfD
# of amino acids	178	265	860	349
S. diarizonae	152/178 (85%)<br>162/178 (91%)	188/258 (73%)<br>214/258 (82%)	690/848 (81%)<br>752/848 (88%)	295/349 (85%)<br>319/349 (91%)
S. enterica subsp. VII	154/178 (87%)<br>161/178 (90%)	168/220 (76%)<br>190/220 (86%)	682/862 (79%)<br>762/862 (88%)	284/346 (82%)<br>313/346 (90%)

* Identity is presented in red (upper line) and similarity is presented black text (bottom line).
