# Supplementary material for: The plasmid-encoded Ipf and Klf fimbriae display different expression and varying roles in the virulence of Salmonella enterica serovar Infantis in mouse vs. avian hosts
Source: PLoS Pathog. 2017 Aug 17;13(8):e1006559. doi: 10.1371/journal.ppat.1006559 (PMC5560535; doi:10.1371/journal.ppat.1006559)
Supplement: S2 Table — (PDF) [file ppat.1006559.s002.pdf]

S2 Table. Homology of the Klf subunits to the *E. coli* K88-Fae fimbria

Protein	Size	Homolog in *E. coli*	Annotation	Accession #	% Identity	% Similarity	E-value
KlfL	44		hypothetical protein	WP_077768209	22/44 (50%)	30/44 (68%)	2e-06
KlfB	106		AFA-III adhesin operon regulatory protein	WP_033555783	60/88 (68%)	74/88 (84%)	2e-39
KlfC	179	FaeC	K88 fimbrial protein A	CUA13245	142/180 (79%)	163/180 (90%)	8e-102
KlfD	772	FaeD	outer membrane usher protein	WP_021564137	670/772 (87%)	717/772 (92%)	0.0
KlfE	261	FaeE	K88 fimbrial chaperone	ANO75879	218/247 (88%)	230/247 (93%)	2e-162
KlfF	164	FaeF	K88 minor fimbrial subunit	WP_000753462	115/162 (71%)	135/162 (83%)	3e-75
KlfG	274	FaeG	fimbrial protein	WP_047613538	97/293 (33%)	143/293 (48%)	8e-39
			fimbrial adhesin	4WEI_A	68/256 (27%)	118/256 (46%)	5e-21
KlfH	263	FaeH	K88 minor fimbrial subunit	WP_001558335	209/262 (80%)	237/262 (90%)	3e-156
KlfI	254	FaeI	K88 minor fimbrial subunit	WP_024009624	174/254 (69%)	200/254 (78%)	1e-126
KlfJ	71	-					
KlfK	192	hypothetical protein	hypothetical protein	WP_024194570	77/184 (42%)	103/184 (55%)	8e-31
KlfA	70	faeA-like family protein	faeA-like family protein	WP_001534886	39/69(57%)	45/69(65%)	3e-19
