# Supplementary material for: The plasmid-encoded Ipf and Klf fimbriae display different expression and varying roles in the virulence of Salmonella enterica serovar Infantis in mouse vs. avian hosts
Source: PLoS Pathog. 2017 Aug 17;13(8):e1006559. doi: 10.1371/journal.ppat.1006559 (PMC5560535; doi:10.1371/journal.ppat.1006559)
Supplement: S3 Table — (PDF) [file ppat.1006559.s003.pdf]

S3 Table. Homology[*] between the *S*. Infantis Klf proteins and other *Salmonella* serovars

S. Infantis pESI	KlfC	KlfD	KlfE	KlfF	KlfG	KlfH	KlfI	KlfJ	KlfK	KlfA
# of amino acids	179	772	261	164	274	263	254	71	192	70
S. Anatum	156/162 (96%)<br>158/162 (97%)	756/772 (98%)<br>764/772 (98%)	261/261 (100%)<br>261/261 (100%)	144/151 (95%)<br>146/151 (96%)	-	252/262 (96%)<br>255/262 (97%)	234/254 (92%)<br>239/254 (94%)	46/54 (85%)<br>49/54 (90%)	176/185 (95%)<br>181/185 (97%)	67/70 (96%)<br>68/70 (97%)
S. Bareilly	156/162 (96%)<br>158/162 (97%)	755/772 (98%)<br>763/772 (98%)	259/261 (99%)<br>259/261 (99%)	150/151 (99%)<br>150/151 (99%)	38/150 (25%)<br>64/150 (42%)	252/262 (96%)<br>256/262 (97%)	235/254 (93%)<br>241/254 (94%)	46/54 (85%)<br>49/54 (90%)	176/185 (95%)<br>181/185 (97%)	67/70 (96%)<br>68/70 (97%)
S. Bredeney	156/162 (96%)<br>158/162 (97%)	750/772 (97%)<br>760/772 (98%)	248/263 (94%)<br>251/263 (95%)	149/151 (99%)<br>149/151 (98%)	-	254/262 (97%)<br>257/262 (98%)	235/254 (93%)<br>240/254 (94%)	45/53 (85%)<br>47/53 (88%)	176/185 (95%)<br>181/185 (97%)	67/70 (96%)<br>68/70 (97%)
S. Schwarzengrund	156/162 (96%)<br>158/162 (97%)	748/772 (97%)<br>761/772 (98%)	247/263 (94%)<br>250/263 (95%)	149/151 (99%)<br>149/151 (98%)	-	254/262 (97%)<br>257/262 (98%)	235/254 (93%)<br>240/254 (94%)	45/53 (85%)<br>47/53 (88%)	176/185 (95%)<br>181/185 (97%)	67/70 (96%)<br>68/70 (97%)
S. Montevideo	156/162 (96%)<br>158/162 (97%)	751/772 (97%)<br>761/772 (98%)	245/263 (93%)<br>249/263 (94%)	149/151 (99%)<br>149/151 (98%)	38/150 (25%)<br>64/150 (42%)	252/262 (96%)<br>255/262 (97%)	236/254 (93%)<br>242/254 (95%)	46/53 (87%)<br>48/53 (90%)	176/185 (95%)<br>181/185 (97%)	65/70 (93%)<br>66/70 (94%)
S. Typhimurium	156/162 (96%)<br>158/162 (97%)	748/772 (97%)<br>759/772 (98%)	248/263 (94%)<br>251/263 (95%)	148/151 (98%)<br>148/151 (98%)	38/150 (25%)<br>64/150 (42%)	252/262 (96%)<br>255/262 (97%)	237/254 (93%)<br>241/254 (94%)	45/53 (85%)<br>47/53 (88%)	176/185 (95%)<br>180/185 (97%)	66/70 (94%)<br>67/70 (95%)
S. Cubana	156/162 (96%)<br>158/162 (97%)	747/772 (97%)<br>761/772 (98%)	239/240 (99%)<br>239/240 (99%)	147/151 (97%)<br>147/151 (97%)	38/150 (25%)<br>64/150 (42%)	253/262 (97%)<br>256/262 (97%)	236/254 (93%)<br>240/254 (94%)	49/55 (89%)<br>50/55 (90%)	176/185 (95%)<br>180/185 (97%)	66/70 (94%)<br>67/70 (95%)

* Identity is presented in red and similarity is presented black text.
