# Supplementary material for: The plasmid-encoded Ipf and Klf fimbriae display different expression and varying roles in the virulence of Salmonella enterica serovar Infantis in mouse vs. avian hosts
Source: PLoS Pathog. 2017 Aug 17;13(8):e1006559. doi: 10.1371/journal.ppat.1006559 (PMC5560535; doi:10.1371/journal.ppat.1006559)
Supplement: S4 Table — (PDF) [file ppat.1006559.s004.pdf]

S4 Table. Bacterial strains utilized in this study

**Strain**	**Description**	**Source or Reference**
S. Infantis 119944	2008 clinical isolate. Sequenced strain	[1]
S. Infantis 119944/pCS26	*S*. Infantis 119944/pCS26	this study
S. Infantis 119944/pCS26::P*ipf*	*S*. Infantis 119944/pCS26::P*ipf*	this study
S. Infantis 119944/pCS26::P*klf*	*S*. Infantis 119944/pCS26::P*klf*	this study
S. Infantis 119944 *ipf*	*ipf* deletion mutant	this study
S. Infantis 119944 *klf*	*klf* deletion mutant	this study
S. Infantis 119944 *klfL*	*klfL* deletion mutant	this study
S. Infantis 119944 *klfB*	*klfB* deletion mutant	this study
S. Infantis 119944 *klfA*	*klfA* deletion mutant	this study
S. Infantis 119944 *fnr*	*fnr* deletion mutant	[2]
S. Infantis 119944 *fur*	*fur* deletion mutant	[2]
S. Infantis 119944 *arcA*	*arcA* deletion mutant	[2]
S. Infantis 119944 *arcB*	*arcB* deletion mutant	[2]
S. Infantis 119944 *phoP*	*phoP* deletion mutant	[2]
S. Infantis 119944 *ompR*	*ompR* deletion mutant	[2]
S. Infantis 119944 *oxyR*	*oxyR* deletion mutant	[2]
S. Infantis 119944 *soxR*	*soxR* deletion mutant	[2]
S. Infantis 119944 *lrp*	*lrp* deletion mutant	[2]
S. Infantis 119944 *rpoS*	*rpoS* deletion mutant	[2]
S. Infantis 119944/pACYC184	*S*. Infantis 119944/pACYC184	this study
S. Infantis 119944/pACYC184-2HA tag::*klfC*	*S*. Infantis 119944/pACYC184-2HA tag::*klfC*	this study
S. Infantis 119944 *fnr*/pACYC184-2HA tag::*klfC*	*S*. Infantis 119944 *fnr*/pACYC184-2HA tag::*klfC*	this study
S. Infantis 119944 *fur*/pACYC184-2HA tag::*klfC*	*S*. Infantis 119944 *fur*/pACYC184-2HA tag::*klfC*	this study
S. Infantis 119944 *arcA*/pACYC184-2HA tag::*klfC*	*S*. Infantis 119944 *arcA*/pACYC184-2HA tag::*klfC*	this study
S. Infantis 119944 *arcB*/ pACYC184-2HA tag::*klfC*	*S*. Infantis 119944 *arcB*/pACYC184-2HA tag::*klfC*	this study
S. Infantis 119944 *phoP*/pACYC184-2HA tag::*klfC*	*S*. Infantis 119944 *phoP*/pACYC184-2HA tag::*klfC*	this study
S. Infantis 119944 *ompR*/pACYC184-2HA tag::*klfC*	*S*. Infantis 119944 *ompR*/pACYC184-2HA tag::*klfC*	this study
S. Infantis 119944 *oxyR*/pACYC184-2HA tag::*klfC*	*S*. Infantis 119944 *oxyR*/pACYC184-2HA tag::*klfC*	this study
S. Infantis 119944 *soxR*/pACYC184-2HA tag::*klfC*	*S*. Infantis 119944 *soxR*/pACYC184-2HA tag::*klfC*	this study
S. Infantis 119944 *lrp*/pACYC184-2HA tag::*klfC*	*S*. Infantis 119944 *lrp*/pACYC184-2HA tag::*klfC*	this study
S. Infantis 119944 *rpoS*/pACYC184-2HA tag::*klfC*	*S*. Infantis 119944 *rpoS*/pACYC184-2HA tag::*klfC*	this study
S. Infantis 119944 *klfA*/pACYC184-2HA tag::*klfC*	*S*. Infantis 119944 *klfA/*pACYC184-2HA tag::*klfC*	this study
S. Infantis 119944 *klfB*/pACYC184-2HA tag::*klfC*	*S*. Infantis 119944 *klfB/*pACYC184-2HA tag::*klfC*	this study

S. Infantis 119944 *klfL*/pACYC184-2HA tag*::klfC*	*S.* Infantis 119944 *klfL/*pACYC184-2HA tag*::klfC*	this study
S. Infantis 119944/pWSK29	*S.* Infantis 119944/pWSK29	this study
S. Infantis 119944/pWSK29-2HA tag*::ipfD*	*S.* Infantis 119944/pWSK29-2HA tag*::ipfD*	this study
S. Infantis 119944 *fnr/*pWSK29-2HA tag*::ipfD*	*S.* Infantis 119944 *fnr/*pWSK29-2HA tag*::ipfD*	this study
S. Infantis 119944 *fur*/pWSK29-2HA tag*::ipfD*	*S.* Infantis 119944 *fur*/pWSK29-2HA tag*::ipfD*	this study
S. Infantis 119944 *arcA*/pWSK29-2HA tag*::ipfD*	*S.* Infantis 119944 *arcA*/pWSK29-2HA tag*::ipfD*	this study
S. Infantis 119944 *arcB*/pWSK29-2HA tag*::ipfD*	*S.* Infantis 119944 *arcB*/pWSK29-2HA tag*::ipfD*	this study
S. Infantis 119944 *phoP*/pWSK29-2HA tag*::ipfD*	*S.* Infantis 119944 *phoP*/pWSK29-2HA tag*::ipfD*	this study
S. Infantis 119944 *ompR*/pWSK29-2HA tag*::ipfD*	*S.* Infantis 119944 *ompR*/pWSK29-2HA tag*::ipfD*	this study
S. Infantis 119944 *oxyR*/pWSK29-2HA tag*::ipfD*	*S.* Infantis 119944 *oxyR*/pWSK29-2HA tag*::ipfD*	this study
S. Infantis 119944 *soxR*/pWSK29-2HA tag*::ipfD*	*S.* Infantis 119944 *soxR*/pWSK29-2HA tag*::ipfD*	this study
S. Infantis 119944 *lrp*/pWSK29-2HA tag*::ipfD*	*S.* Infantis 119944 *lrp*/pWSK29-2HA tag*::ipfD*	this study
S. Infantis 119944 *rpoS*/pWSK29-2HA tag*::ipfD*	*S.* Infantis 119944 *rpoS*/pWSK29-2HA tag*::ipfD*	this study
E. coli ORN172	*thr-l leuB thi-1* Δ*(argF-lac)U169 xyl-7 ara-13 mtl-2 gal-6 rpsL tonA2 supE44* Δ*(fimBEACDFGH)::kan pilG1*	[3]
E. coli ORN172/ pBAD18	*E. coli* ORN172/pBAD18	this study
E. coli ORN172/ pBAD18*::ipf*	*E. coli* ORN172/pBAD18*::ipf*	this study
E. coli ORN172/ pBAD18*::klf*	*E. coli* ORN172/pBAD18*::klf*	this study
E. coli ORN172/ P_{tetA}-*ipfABCD*	*E. coli* ORN172/pWSK29::P_{tetA}-*ipfABCD*	this study
E. coli ORN172/ p4391	*E. coli* ORN172 pWSK29::P_{tetA}-*stbABCDEFG*	[4]
S. Infantis 119944/ pWSK29	*S.* Infantis 119944/pWSK29	this study
S. Infantis 119944/ pWSK129	*S.* Infantis 119944/pWSK129	this study
S. Infantis 119944/ *ipf*/pWSK29	*ipf* deletion mutant/pWSK29	this study
S. Infantis 119944/ *klf*/pWSK29	*klf* deletion mutant/pWSK29	this study
**Plasmids**		
pKD46		[5]
pKD3		[5]
pCP20		[5]
pWSK129	Km^r low copy number cloning vector	[6]
pWSK29	Amp^r low copy number cloning vector	[6]
pWSK29-2HA tag	2HA tag cloned into pWSK29	
pWSK29-2HA tag*::ipfD*	*S.* Infantis *ipfD* fused to 2HA tag cloned into pWSK29	this study
pWSK29::P_{tetA}-*ipfABCD*	*S.* Infantis *ipfABCD* cloned under *tet* inducible promoter	this study
pACYC184	Tet^r Cm^r cloning vector	(NEB)
pACYC184-2HA tag	2HA tag cloned into pACYC184	

pACYC184-2HA tag::*klfC*	*S*. Infantis *klfC* fused to 2HA tag cloned into pACYC184	this study
pCS26	Kan^r cloning vector for *luxCDABE* fusion	[7]
pCS26::P*ipf*	*S*. Infantis *ipf* regulatory region cloned into pCS26	this study
pCS26::P*klf*	*S*. Infantis *klf* regulatory region cloned into pCS26	this study
pBAD18	Arabinose-inducible expression plasmid, Amp^r	[8]
pBAD18::*ipf*	*ipfABCD* fimbria cloned into pBAD18	this study
pBAD18::*klf*	*klfBCDEFGHIJKA* fimbria cloned into pBAD18	this study
p4392	*S*. Typhimurium type 1 fimbriae cloned under tetracycline inducible promoter; amplification of vector for Gibson assembly with *ipfABCD*	[4]
p4391	pWSK29::P$_{tetA}$-*stbABCDEFG*	[4]

References

1. Aviv G, Tsyba K, Steck N, Salmon-Divon M, Cornelius A, Rahav G, Grassl GA & Gal-Mor O. A unique megaplasmid contributes to stress tolerance and pathogenicity of an emergent Salmonella enterica serovar Infantis strain. Environmental microbiology, 16:977-994 (2014).
2. Aviv G, Rahav G & Gal-Mor O. Horizontal Transfer of the *Salmonella enterica* Serovar Infantis Resistance and Virulence Plasmid pESI to the Gut Microbiota of Warm-Blooded Hosts. mBio, e01395-16. doi: 10.1128/mBio.01395-16 (2016).
3. Woodall LD, Russell PW, Harris SL, Orndorff PE. 1993. Rapid, synchronous, and stable induction of type 1 piliation in Escherichia coli by using a chromosomal lacUV5 promoter. Journal of bacteriology, 175:2770-2778.
4. Hansmeier N., Miskiewicz K., Elpers L., Liss V., Hensel M., Sterzenbach T. Function expression of the adhesiome of *Salmonella enterica* serotype Typhimurium. (2017, in revision)
5. Datsenko, K.A. & Wanner, B.L. One-step inactivation of chromosomal genes in Escherichia coli K-12 using PCR products. *Proceedings of the National Academy of Sciences of the United States of America* 97, 6640-6645 (2000).
6. Wang, R.F. & Kushner, S.R. Construction of versatile low-copy-number vectors for cloning, sequencing and gene expression in *Escherichia coli*. *Gene* 100, 195-199 (1991).
7. Bjarnason J, Southward CM, Surette MG. Genomic profiling of iron-responsive genes in *Salmonella enterica* serovar Typhimurium by high-throughput screening of a random promoter library. J Bacteriol 185:4973-4982 (2003).
8. Guzman LM, Belin D, Carson MJ, Beckwith J. Tight regulation, modulation, and high level expression by vectors containing the arabinose pBAD promoter. J Bacteriol 177(14):4121-30 (1995).
