# Supplementary material for: The plasmid-encoded Ipf and Klf fimbriae display different expression and varying roles in the virulence of Salmonella enterica serovar Infantis in mouse vs. avian hosts
Source: PLoS Pathog. 2017 Aug 17;13(8):e1006559. doi: 10.1371/journal.ppat.1006559 (PMC5560535; doi:10.1371/journal.ppat.1006559)
Supplement: S5 Table — (PDF) [file ppat.1006559.s005.pdf]

**S5 Table. Primers used in this study**

| Primer name | Sequence (5' to 3') * | Purpose |
|---|---|---|
| RT faeC Fw | GTCATGTGGTGGTGGATGCTG | RT-PCR of *klfC* |
| RT faeC Rv | CAAAATTCAGGGTGGCGATCTG | |
| RT k88 Fw | GCGTCATACGGGTATGGTCT | RT-PCR of *klfD* |
| RT k88 Rv | GGTACTTCGCCGTGGCATAC | |
| RT faeE Fw | GCTGCGTATCATCATGGCCTC | RT-PCR of *klfE* |
| RT faeE Rv | CTTTACGGTCCTTCAGCAGC | |
| RT faeG Fw | GGTGTTGGAGCTGGATTTGATAG | RT-PCR of *klfG* |
| RT faeG Rv | GAACTGGATTTGCGGAGCAGC | |
| RT ipfA Fw | GCGTTTTCAGGCGGAGTTCAG | RT-PCR of *ipfA* |
| RT ipfA Rv | GCAGTCTTGGCAGTAGTGTCC | |
| RT ipfB Fw | CGTTAGTTCAGGCATGGCTTG | RT-PCR of *ipfB* |
| RT ipfB Rv | GATAAAACACCGACTCCCTGTC | |
| RT ipfC Fw | CAGGCAGGGTACAGTAACG | RT-PCR of *ipfC* |
| RT ipfC Rv | GGATAACAGCGAACTCTGAG | |
| RT ipf lectin Fw | CTTGGGCAGTATGACGGACAAG | RT-PCR of *ipfD* |
| RT ipf lectin Rv | CATCTGCCATTTGGAGGAATCACAT | |
| RT 16s rRNA Fw | GGTTAAGTCCCGCAACGAG | RT-PCR of 16S rRNA |

| | | |
|---|---|---|
| RT 16s rRNA Rv | CTTCTCTTTGTATGCGCCATTG | |
| k88 promoter XhoI Fw | TTTTCTCGAGCGCTGCATTAATACCACCAC | cloning of *klf* promoter into pCS26 |
| k88 promoter BamHI Rv | TTTTGGATCCCATTTCTCCCGGAATGAGGG | |
| ipf promoter XhoI Fw | TTTTCTCGAGATAACAATCCACTTTTGTACCATCAC | cloning of *ipf* promoter into pCS26 |
| ipf promoter BamHI Rv | TTTTGGATCCCATACACCAGCTCCTCAATAAC | |
| pCS26 seq Fw | CCGACGTCTAAGAAACCATTATTATC | sequencing of pCS26 inserts |
| pCS26 seq Rv | CACTAAATCATCACTTTCGGGAAAG | |
| faeL Fw | GTGGTGGTATTAATGCAGCG | *klfL* deletion mutant |
| cm 5-faeL (Rv) | GAAGCAGCTCCAGCCTACACACTGGACTATCTGGCGCTTCAT | |
| faeL Rv | GACACCTGTATAGATGGTAGC | |
| cm 3-faeL (Fw) | CTAAGGAGGATATTCATATGCCAGAACGATATGGACAGTG | |
| faeA Fw | GCGGACTCTGATATCCCTGTG | *klfA* deletion mutant |
| cm 5-faeA (Rv) | GAAGCAGCTCCAGCCTACACAGTTCTCCTGTATCAACGGGC | |
| faeA Rv | TCGTCATCCGGCATGTTGTC | |
| cm 3-faeA (Fw) | CTAAGGAGGATATTCATATGCCGATATCCGGTAAAACAAGGG | |
| faeB Fw | GTGGTATTAAAGATCGTGTATTTTG | *klfB* deletion mutant |
| cm 5-faeB (Rv) | GAAGCAGCTCCAGCCTACACACATTTCTCCCGGAATGAGGG | |
| faeB Rv | GGTCTCATACAGCCGCACG | |
| cm 3-faeB (Fw) | CTAAGGAGGATATTCATATGGATAATGAGATAACTGGTCTTTATTTC | |
| k88 fae ko Fw | GTTCTCTCCATGCCCTTGTTTTACCGGATATCGGTCAGACCAGCCGCCATCGGTGTAGGCTGGAGCTGCTTCG | *klf* deletion mutant |
| k88 fae ko Rv | ATCGTTCTGGCTCCGCGACGCAGGGGAGTGCGGGTTACTTTTTTTCCTTTTCCATATGAATATCCTCCTTA | |
| k88 fae ko check Fw | CATCTTCAGAAAGTTCATCAGTC | |

| | | |
|---|---|---|
| k88 fae ko check Rv | GAATTCTTTACCGGTAACGTTAG | |
| fim ko Fw | AAATACTGTTGAGCAGACCTGGATTTCCATGTTCTTATTGATATGAGAATGTGTGTAGGCTGGAGCTGCTTCG | *ipf* deletion mutant |
| fim ko Rv | GCCGGAGATGTTGCTGCTACTGTGAACTACACGATTGCATACGAATAATTATGTCCATATGAATATCCTCC | |
| fim ko check Rv | CAATAATACTTTCTGTCAGATAAC | |
| fim chaperone Fw | GTATCAATCCGGGCATGATAAC | |
| Cm 5 | TGTGTAGGCTGGAGCTGCTTC | chloramphenicol cassette creation |
| Cm 3 | CATATGAATATCCTCCTTAG | |
| faeC SalI 2HA tag Fw | TTTTGTCGACGGATTATGCTTAATAATTTACTTAGGCATAAATATTG | cloning of *klfC* into pACYC184 -2HA tag |
| faeC BglII 2HA tag Rv | TTTTAGATCTCTGATACGTCACCACAAAGG | |
| pACYC184 seq Fw | CACCGGAAGGAGCTGACTG | sequencing inserts of pACYC184 |
| pACYC184 seq Rv | GTAGCACCTGAAGTCAGCCC | |
| ipf lectin Fw for 2HA | TTTTGAGCTCATTATTAACATTCCTAAATAATACGTGGCCCT | cloning of *ipfD* into pWSK29-2HA tag |
| ipf lectin Rv for 2HA | TTTTTCTAGATTGATATGAGAATGTTATTGTTGATACAGCC | |
| M13/Puc primer Fw | GTTTTCCCAGTCACGACGTTG | sequencing inserts of pWSK29 |
| M13/Puc primer Rv | AGCGGATAACAATTTCACACAGGA | |
| | | |
| ipf pBAD Fw | TTTTGAGCTCCTTTCTGTCAGATAACTCCTGGTTG | cloning of *ipf* under P*ara* into pBAD18 |
| ipf pBAD Rv | TTTTTCTAGATTATTGATATGAGAATGTTATTGTTGATACAGCC | |

| | | |
|---|---|---|
| fae-k88 pBAD Fw | TTTTGCTAGCCAGCTTTTATTTCTGGGGTACCG | cloning of *klf* under P*ara* into pBAD18 |
| fae-k88 pBAD Rv | TTTTGAGCTCCCTTGTTTTACCGGATATCGGTCA | |
| fur SalI Fw | TTTTGTCGACCTGCGCCGCATCAATAGAC | cloning of *fur* into pACYC184 |
| fur HindIII Rv | TTTTAAGCTTTTATTTAGTCGCGTCATCGTGC | |
| lrp sacI clone Fw | TTTTGAGCTCGCGACAGCGGCGTTATCATC | cloning of *lrp* into pWSK29 |
| lrp xbaI clone Rv | TTTTTCTAGAAACTACGGCGATTTTGCACC | |
| 1f-SIN-ipf | TGCATCAAAAAAACCAGGTAATGATTCTGTTATTGAGGAGCTGG TGTA | cloning of *ipf* under P*tet* into pWSK29 |
| 1r-SIN-ipf | CCCGGGCTGCAGGAATTCCCATCTCTTATTACTTACTCGATATTC CTGA | |
| Vf-pWSK29-Ptet | GAATTCCTGCAGCCCGGGG | Amplification of vector containing *aph tetR* $P_{tetA}$ |
| Vr-pWSK29-Ptet | CATTACCTGGTTTTTTTGATGCATTTCACT | |

* Nucleotide sequence from lambda-red recombinase antibiotic resistant cassettes and restriction enzyme sites added to the primers are underlined.
