# Supplementary figures and images for: The plasmid-encoded Ipf and Klf fimbriae display different expression and varying roles in the virulence of Salmonella enterica serovar Infantis in mouse vs. avian hosts
Source: PLoS Pathog. 2017 Aug 17;13(8):e1006559. doi: 10.1371/journal.ppat.1006559 (PMC5560535; doi:10.1371/journal.ppat.1006559)

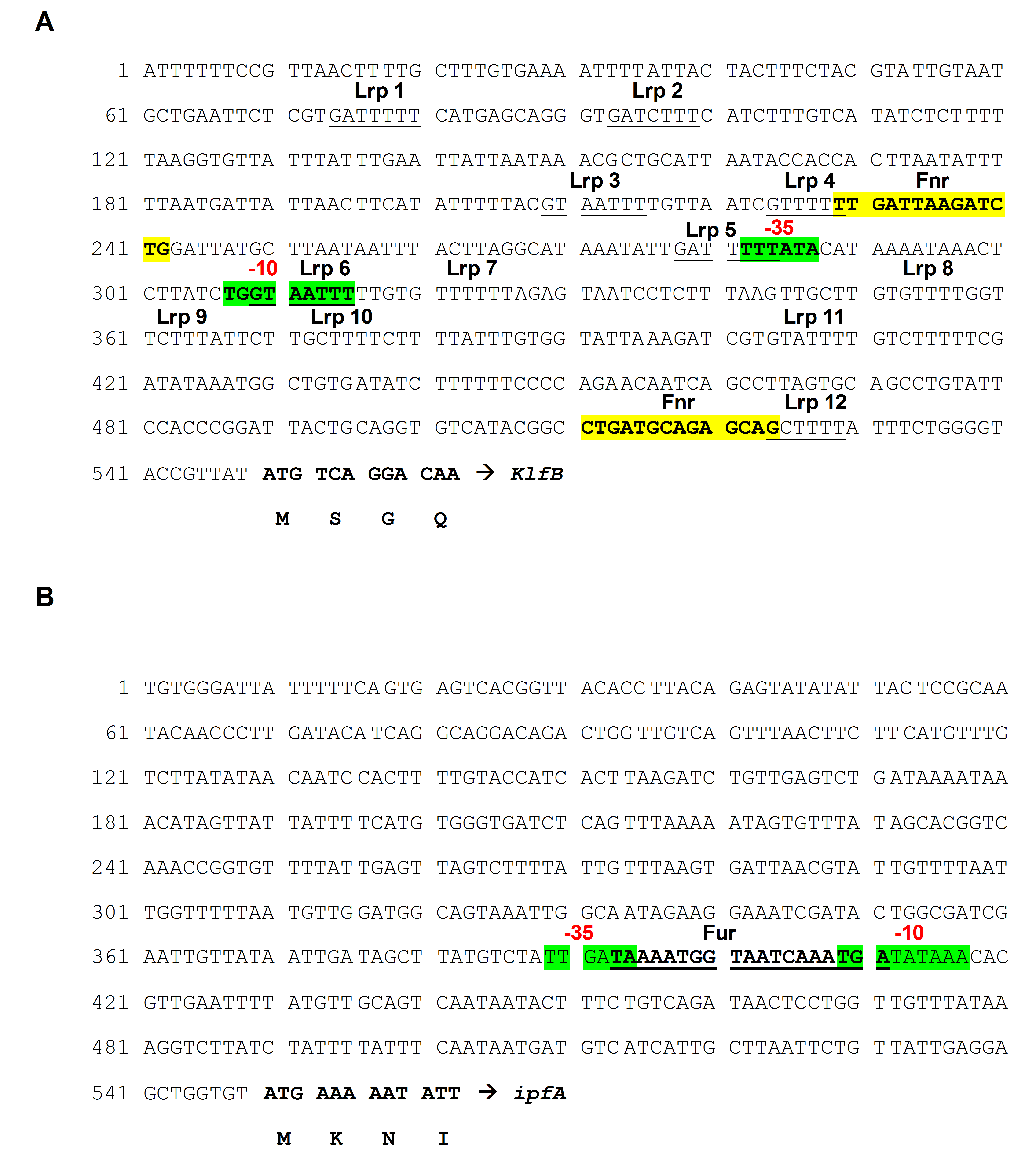

Supplement: S1 Fig — DNA sequences containg 574 bp upstream from klfB and ipfA were analysed in-silico. Promoter location including the -10 and -35 boxes was predicted by BPROM, and the Fnr and Fur binding sites were predicted by the Virtual Footprint tool. (A) The klfB promoter region. -10 and -35 sites are highlighted in green and indicated by red text, predicted Fnr binding sites are in bold font and highlighted in yellow. Twelve putative Lrp binding sites (Lrp1-12) are numbered and underlined. (B) The ipfA promoter region. -10 and -35 sites are highlighted in green and the predicted Fur binding site is in bold font and underlined. (TIF) [file ppat.1006559.s006.tif]

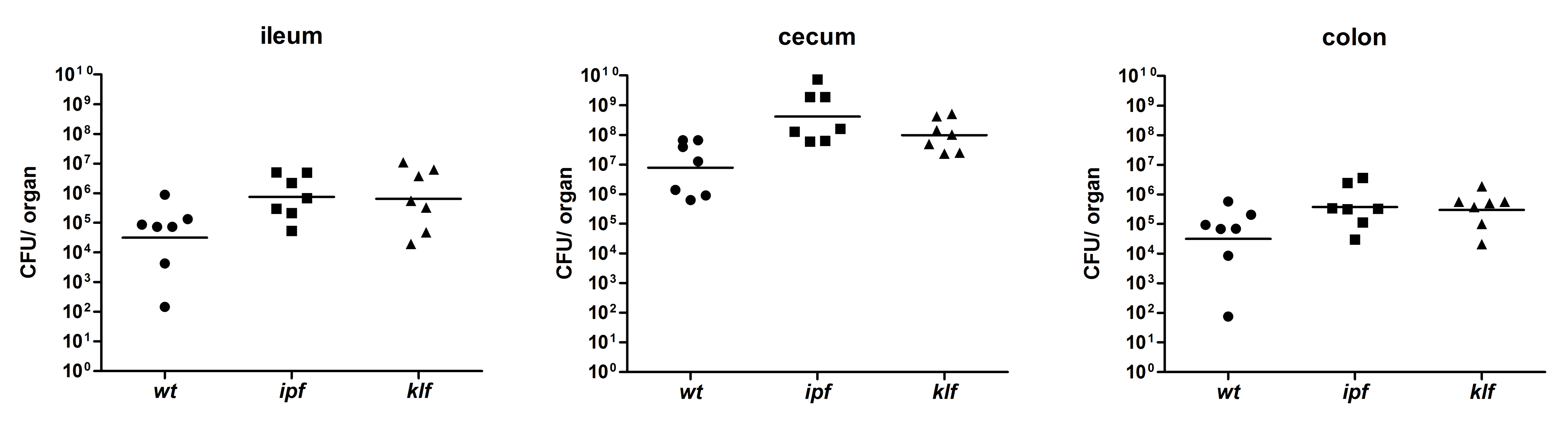

Supplement: S2 Fig — Five days p.i. chicks were sacrificed and the indicated organs were homogenized and plated on XLD agar plates supplemented with tetracycline for bacterial numeration. Bacterial burden in the cecum, ileum and colon is shown. Each dot represents the count in a single entire organ. The geometrical mean in each organ is shown by a solid horizontal line. (TIF) [file ppat.1006559.s007.tif]
